# Influence of cigarette smoking on the human duodenal mucosa-associated microbiota

**DOI:** 10.1186/s40168-018-0531-3

**Published:** 2018-08-29

**Authors:** Erin R. Shanahan, Ayesha Shah, Natasha Koloski, Marjorie M. Walker, Nicholas J. Talley, Mark Morrison, Gerald J. Holtmann

**Affiliations:** 1 Department of Gastroenterology and Hepatology, Princess Alexandra Hospital, and Faculty of Medicine, The University of Queensland, 199 Ipswich Road, Woolloongabba, Brisbane, Queensland 4102 Australia; 2 Translational Research Institute, Woolloongabba, Queensland Australia; 3 Faculty of Medicine, The University of Queensland Diamantina Institute, The University of Queensland, Saint Lucia, Queensland 4072 Australia; 4 Faculty of Health and Medicine, University of Newcastle, Callaghan, New South Wales Australia; 5 Present address: School of Life and Environmental Sciences, Charles Perkins Centre, The University of Sydney, Camperdown, New South Wales Australia

**Keywords:** Microbiome, Small intestine, Duodenum, Mucosa, Smoking, Cigarettes

## Abstract

**Background:**

Cigarette smoking is a known risk factor in a number of gastrointestinal (GI) diseases in which the microbiota is implicated, including duodenal ulcer and Crohn’s disease. Smoking has the potential to alter the microbiota; however, to date, the impact of smoking on the mucosa-associated microbiota (MAM), and particularly that of the upper GI tract, remains very poorly characterised. Thus, we investigated the impact of smoking on the upper small intestinal MAM. A total of 102 patients undergoing upper GI endoscopy for the assessment of GI symptoms, iron deficiency, or Crohn’s disease, but without identifiable lesions in the duodenum, were recruited. Smoking status was determined during clinical assessment and patients classified as current (*n* = 21), previous smokers (*n* = 40), or having never smoked (*n* = 41). The duodenal (D2) MAM was profiled via 16S rRNA gene amplicon sequencing.

**Results:**

Smoking, both current and previous, is associated with significantly reduced bacterial diversity in the upper small intestinal mucosa, as compared to patients who had never smoked. This was accompanied by higher relative abundance of *Firmicutes*, specifically *Streptococcus* and *Veillonella* spp*.* The relative abundance of the genus *Rothia* was also observed to be greater in current smokers; while in contrast, levels of *Prevotella* and *Neisseria* were lower. The MAM profiles and diversity of previous smokers were observed to be intermediate between current and never smokers. Smoking did not impact the total density of bacteria present on the mucosa.

**Conclusions:**

These data indicate the duodenal MAM of current smokers is characterised by reduced bacterial diversity, which is partially but not completely restored in previous smokers. While the precise mechanisms remain to be elucidated, these microbiota changes may in some part explain the adverse effects of smoking on mucosa-associated diseases of the GI tract. Smoking status requires consideration when interpreting MAM data.

**Electronic supplementary material:**

The online version of this article (10.1186/s40168-018-0531-3) contains supplementary material, which is available to authorized users.

## Background

Cigarette smoking can modulate both the risk and clinical course of a number of gastrointestinal (GI) disorders including inflammatory bowel disease [1], irritable bowel syndrome [2], peptic ulcer disease [3], and GI cancer [4, 5]. Overall, smoking is now considered the most important environmental factor affecting the recurrence of Crohn’s disease (CD) [6]. Cigarette smoking also alters the risks associated with GI infections, most notably *Helicobacter pylori* infection [7].

Many of these disorders are also associated with alterations to the mucosal microbiota [8–10]. The microbes colonising the GI mucosa aid in promoting gut health and immune homeostasis, and changes to the community composition and/or density of these microbes are implicated in a variety of disease states. Cigarette smoking is an environmental factor which may influence the composition of the microbiota [11–13]. Cigarette smoke is a source of multiple toxicants and has the potential to influence the microbiota via changes to immune homeostasis [14], mucin production [15], oxygen tension [16], or through direct antimicrobial effects [17]. It can also alter GI physiology through altering duodenal pH [18], reducing pancreatic bicarbonate secretions [19], and altering gastric emptying time [20].

Recent evidence implicates the small intestinal microbiota, and particularly the mucosa-associated microbiota (MAM), as an important modulator of GI health [21]. Changes to the small intestinal microbiota have been observed in coeliac disease [22, 23], diabetes mellitus [24], chronic liver disease [25], irritable bowel syndrome [26], and functional dyspepsia [27]. Given the potential for cigarette smoking to exert influence over both the gut environment and the microbiota itself, cigarette smoking is likely to have important implications regarding the host-microbe interactions, and various gut disorders, associated with the small intestine. Thus, smoking may represent an important confounding factor in understanding the MAM. However, the impact of cigarette smoking on the small intestinal MAM has not been well-studied and remains very poorly understood.

With this background, we hypothesised that cigarette smoking is associated with alterations to the MAM in the small intestine. We aimed to compare the bacterial community composition and diversity in the upper small intestinal MAM in patients undergoing routine endoscopy, who were current cigarette smokers, had never smoked, or who had discontinued smoking.

## Methods

### Patient recruitment and sample collection

Ethics approval was obtained by the responsible institutional review board (Metro South Health) and patients recruited at the outpatients clinic of the Department of Gastroenterology and Hepatology at the Princess Alexandra Hospital, Brisbane, Australia. We included patients presenting with documented iron deficiency (ID, with and without anaemia), functional dyspepsia (FD) or FD with additional irritable bowel syndrome-like symptoms based on Rome IV [28] (Additional file 1: Table S1), or Crohn’s disease (CD, Additional file 1: Table S2). Patients with antibiotic use up to 2 months prior to endoscopy were excluded. One hundred and two patients, all of whom did not show any evidence of gastric/duodenal mucosal abnormalities, lesions, or structural changes (based upon endoscopic and clinical histology findings), were included. Intestinal biopsies were taken from the 2nd part of the duodenum utilising the Brisbane Aseptic Biopsy device (MTW, Germany) [29], which enables specific sampling of the MAM, through collection of mucosal samples with exclusion of contamination from luminal contents or other regions of the GI tract. Biopsy samples were immediately placed under aseptic conditions into a sterile tube containing RNAlater (Qiagen). Samples were allowed to incubate at room temperature for 30 min then frozen and stored at − 80 °C.

### DNA extraction

Samples were lysed using a protocol optimised for extraction of microbial DNA for community analyses [30]. Frozen samples were thawed on ice, and individual tissue biopsy samples (approx. 1–2 mm^3^) were removed from the RNAlater and the tissue utilised for gDNA extraction. Each sample was placed in a screw-cap tube containing 300 μL lysis buffer (NaCl 0.5 M, Tris-HCl 50 mM,pH 8.0, EDTA 50 mM, and SDS 4% *w*/*v*) and 0.4 g sterile zirconia beads (1:1, 0.1 mm, and 1 mm; Daintree Scientific). Homogenisation was undertaken in a tissue homogeniser (Precellys) for 3 min followed by incubation at 70 °C for 10 min. The lysate was collected and the homogenisation procedure repeated with the addition of further lysis buffer, providing 500 μL of pooled lysate for each sample to be used for DNA extraction. The DNA was extracted using an automated system (Maxwell® 16) with the Maxwell® 16 Tissue DNA Purification Kit (Promega), following the manufacturer’s instructions. Extracted gDNA was quantified (Nanodrop) and stored at − 80 °C.

Recent studies have demonstrated that samples with relatively small amounts of microbial biomass can produce spurious results, due in part to DNA contamination of the reagents used [31]. To assess the possible impact of this on our results, we also prepared a set of reagent controls, to which no additional tissue or DNA was added. These reagent only mixtures were processed in an identical manner to the tissue samples, commencing at the lysis step.

### Assessment of bacterial load

Bacterial load in samples was assessed through quantitative PCR (qPCR). As the gDNA extracted from the biopsy samples consists of a mixture of human and bacterial DNA, both the human β-actin gene and bacterial 16S rRNA gene were assessed. The following optimised primer sets were utilised: β-actin (forward: TCCGCAAAGACCTGTACGC; reverse: CAGTGAGGACCCTGGATGTG) and bacterial domain specific 16S rRNA (1114-forward: CGGCAACGAGCGCAACCC; 1221-reverse: CCATTGTAGCACGTGTGTAGCC). Standards of known copy number were constructed using serial dilutions of the pUC19 plasmid with the human β-actin or *Streptococcus* spp. 16S rRNA PCR product inserted. The Power SYBR Green Master Mix (Life Technologies) was used and samples analysed on a ViiA 7 Real-Time PCR system. Both the reagent controls (described above) and template-free samples were used as negative controls. The number of β-actin- and bacterial 16S rRNA-encoding gene copies in each sample were quantified with respect to their standard curves, and bacterial load was calculated from the ratio between 16S rRNA genes:β-actin genes. Statistical analysis of results was performed in Prism, with differences assessed via Mann-Whitney, Kruskal-Wallis, or Spearman correlation as appropriate.

### Library preparation and sequencing

The small intestinal and control samples were profiled by high-throughput amplicon sequencing with dual-index barcoding using the Illumina MiSeq platform. The V6-V8 region of the gene encoding 16S ribosomal RNA was amplified using the primers 917-forward (GAATTGRCGGGGRCC; bacterial domain specific) and 1392-reverse (ACGGGCGGTGWGTRC; universal), which also contained Illumina adapter sequences. Amplification was undertaken using the Q5 DNA polymerase (NEB) as per the manufacturer’s instructions. PCR products were purified using AMPure XP beads (Beckman Coulter). The PCR libraries were then barcoded using the Illumina dual-index system (Nextera XT v2 Index Kit). Following a second round of purification (AMPure XP beads), libraries were quantified (Quantus) and pooled to 4 nM. The libraries were sequenced on an Illumina MiSeq using the MiSeq Reagent Kit v3 (2 × 300 bp), using facilities provided by the Australian Centre for Ecogenomics.

### Bioinformatics

Sequence data was processed using the Quantitative Insights Into Microbial Ecology (QIIME) pipeline (version 1.9.1) [32]. Further details of the workflow utilised are provided in the Additional file 2. Briefly, the split_libraries_fastq.py command was applied using a Phred quality threshold of Q20. Operational taxonomic units (OTUs) were assigned using the pick_open_reference_otus.py command [33]. The Greengenes database (version 13.8) was used as the reference database and a sequence similarity of 97% applied [34]. The resulting OTU table was chimera-checked using ChimeraSlayer [35] and subsequently filtered to remove sequences with a relative abundance of less than 0.1%. The reagent control samples were concurrently processed to generate a list of specific “contaminant” OTUs (Additional file 2). These specific OTUs were then filtered from the small intestinal samples to generate an OTU table which represented contamination-free small intestinal sequences. All samples with a final read count of less than 1000 sequence reads were also excluded from the OTU table (Additional file 3: Table S3).

### Statistical analyses

To assess bacterial diversity (alpha-diversity), rarefaction curves were generated on the raw OTU table and a read depth of 1000 reads was selected for analysis of diversity. The OTU table was randomly sub-sampled 100 times and diversity assessed using the Chao1 [36] and Faith’s phylogenetic diversity (whole tree) [37] metrics, as implemented with the alpha_rarefaction.py workflow script in QIIME. Significance was tested using Kruskal-Wallis. Multivariate analysis (linear regression model) was performed via ANOVA.

To assess relative abundance of bacterial taxa, the OTU table was normalised via total sum scaling, followed by centred-log ratio transformation. Average relative abundances were calculated and significant differences assessed using Kruskal-Wallis (KW) with false discovery rate (FDR) correction for multiple comparisons. A *p* value of < 0.05 and FDR *q* value of < 0.05 was considered significant. In order to confirm differences in OTU relative abundance and further avoid potential issues with compositional data [38, 39], the ALDEx2 function [40], implemented in the microbiome analysis tool Calypso [41], was utilised on non-normalised data. Multivariate analysis (linear regression model) was performed via ANOVA.

To assess beta-diversity and the relationship between smoking status and the overall bacterial community, weighted and unweighted UniFrac, along with Bray-Curtis, distance matrices were constructed. The OTU table was normalised via random sub-sampling 100 times to a depth of 1000 sequence reads [42]. The distance matrix was then generated using the beta_diversity_through_plots.py script as implemented in QIIME. Principal coordinate plots were generated from the distance matrix using the first two coordinates and coded based on smoking status and patient diagnosis. To test differences in overall community composition based on smoking status, the ADONIS [43] permutational MANOVA, as implemented in Calypso [41], was used, adjusting for sex, age, body mass index (BMI), proton pump inhibitor (PPI) use, and patient diagnosis.

Linear discriminant analysis effect size (LEfSe) [44] was used to identify taxa associated with smoking status. This was performed on the OTU table normalised by total sum scaling and centred-log ratio transformation, as implemented in Calypso [41].

To generate a constrained multivariate model and to differentiate current smokers from those individuals who had never smoked, the sparse partial least squares discriminant analysis method (MixMC [45]) was utilised. The OTU table was filtered to include only samples from patients with FD or ID, only current and never smokers, and normalised by total sum scaling followed by centred-log ratio transformation [45]. This data was used as the training set to generate the model. The model was validated using the leave-one-out validation method. The model was then tested on a “test” data set consisting of the CD patients (OTU table normalised as above). A plot was also generated based on the first two components of the model, following prediction of smoking status, on both the training and test sets.

## Results

### Study cohort

A total of 102 patients were recruited into the study. Smoking status was determined during clinical assessment of patients and subjects grouped as current smokers (*n* = 21), previous smokers (*n* = 40), and persons who had never smoked (*n* = 41) (Table 1). There was no significant difference in smoking status when patients were grouped according to diagnosis (FD, ID, or CD); however, there were no current smokers in the iron deficiency screening cohort. Additionally, there were no significant differences in the sex distribution, PPI use, or BMI across smoking status; however, former smokers were significantly older than current or never smokers (Table 1).

**Table 1:** Patient cohort characteristics

	Current	Previous	Never	*p* value^a^	Overall cohort
Functional dyspepsia/iron deficiency					
Female, *n* (%)	7 (50)	20 (60.6)	15 (42.9)	0.34^b^	42 (51.2)
Age, median (range)	49.1 (28.3–65.5)	62 (20.1–78.6)	45.8 (17.2–77.5)	0.02^c,d^	54.7 (17.2–78.6)
BMI, mean (SD)	28.6 (7.6)	27.1 (5.5)	27.1 (6.2)	0.8^e^	27.3 (6.2)
Patient—Functional/iron deficiency, *n* (%)	14 (100)/0 (0)	27 (81.8)/6 (18.2)	26 (74.3)/9 (25.7)	0.11^b^	67 (81.7)/15 (18.3)
Current PPI use, *n* (%)	8 (57.1)	19 (57.6)	19 (54.3)	0.84^b^	46 (56.1)
Country of birth—Australia/others, *n* (%)	9 (64.3)/5 (35.7)	19 (57.6)/14 (42.4)	20 (57.1)/15 (42.9)	0.89^b^	48 (58.5)/34 (41.5)
Crohn’s disease					
Female, *n* (%)	5 (71.4)	4 (57.1)	5 (83)	0.59^b^	14 (70)
Age, median (range)	36.4 (22.2–57.9)	50.2 (37.5–59.0)	47.5 (22.2–61.9)	0.34^c^	45.6 (22.2–61.9)
BMI, mean (SD)	30.2 (7.3)	32.2 (6.9)	26.7 (5.4)	0.46^e^	30.0 (7.0)
Current PPI use, *n* (%)	0 (0)	0 (0)	3 (50)	0.07^f^	3 (15)
Country of birth—Australia/others, *n* (%)	7 (100)/0 (0)	6 (85.7)/1 (14.3)	3 (50)/3 (50)	0.07^b^	16 (80)/4 (20)

^a ^Between smoking status. ^b^ *p* values based on chi-squared test. ^c^ *p* values based on Kruskal-Wallis test. ^d^ On Dunn’s multiple comparison test, only Previous vs Never was significantly different (*p* = 0.03). ^e^ *p* values based on ANOVA. ^f^ *p* values based on Fisher’s exact test. BMI—body mass index. PPI—proton pump inhibitor

### Smoking does not alter small intestinal bacterial load

**Fig. 1:**
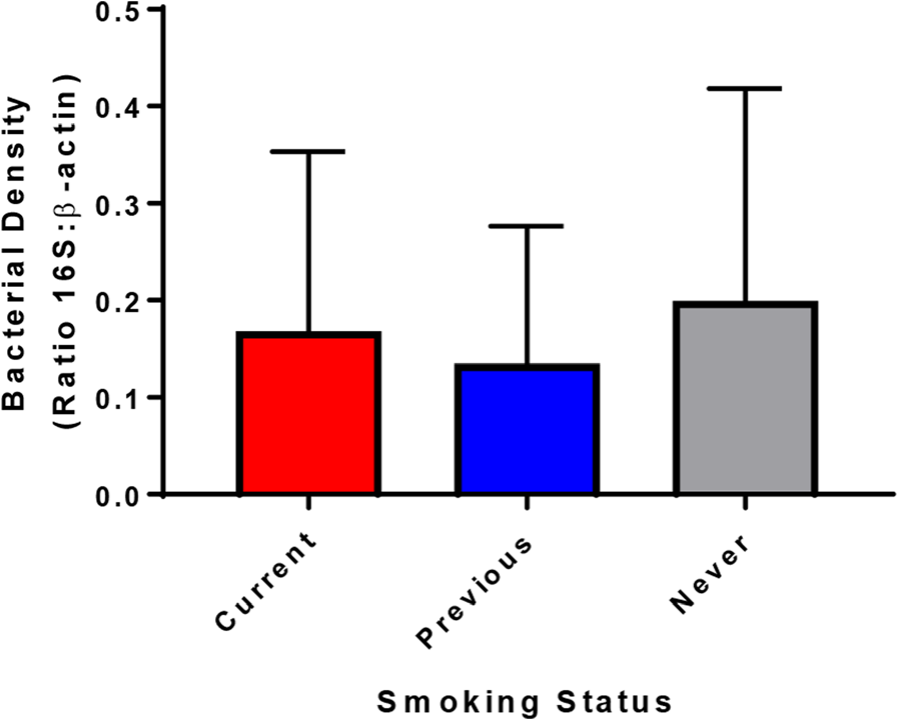
Bacterial load on the small intestinal mucosa in all patients. Load was assessed by qPCR and expressed as the ratio between copies of the bacterial 16S rRNA gene and copies of the human beta-actin gene. Significance testing was undertaken using Kruskal-Wallis; in addition, a multivariate analysis was undertaken in which patient diagnosis, age, sex, BMI and PPI use were included. In both cases, no significant differences in bacterial load were observed based on smoking status

The density of bacteria adherent to the small intestinal mucosa was assessed via qPCR. There was no significant difference in bacterial load observed between current, previous, and never smokers (Fig. 1, Additional file 1: Figure S1).

### Small intestinal bacterial diversity is lower in smokers

**Fig. 2:**
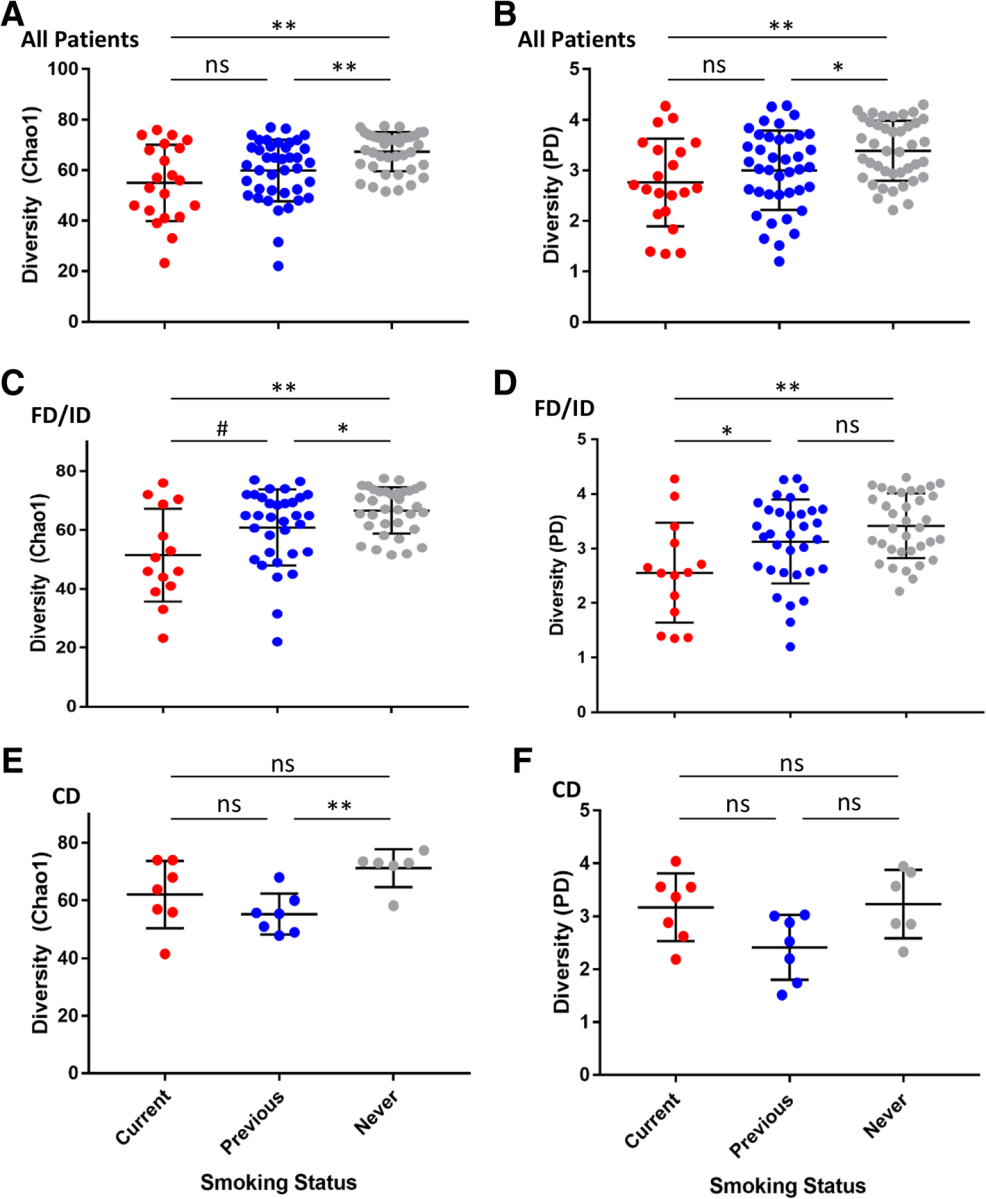
Bacterial diversity within the mucosa-associated microbiota from duodenal (2nd part) biopsies. Patients were grouped based on smoking status (current smokers, previous smokers, and having never smoked). The **a**, **c**, and **e** Chao1 index and **b**, **d**, and **f** Faith’s phylogenetic diversity (PD) index of diversity within samples was then calculated for **a**, **b** all patients, **c**, **d** FD/ID and **e**, **f** CD patients. Mean and standard deviation are shown. ns—not significant, # *p* = 0.05, * *p* < 0.05, ** *p* < 0.01 Kruskal-Wallis. CD—Crohn’s disease; FD—functional dyspepsia; ID—iron deficiency

The composition of the upper small intestinal MAM was established via 16S rRNA gene amplicon sequencing. Overall, the alpha (within sample) diversity of the MAM was significantly reduced in current smokers compared to those individuals who have never smoked (Fig. 2a, b). In addition, previous smokers were found to have significantly reduced MAM diversity compared to never smokers. This was observed in both the Chao1 index, as well as using Faith’s phylogenetic diversity which accounts for the phylogenetic relationships between the organisms observed. Patient age, sex, BMI, PPI use, and diagnosis did not significantly impact diversity, and the significantly lower diversity in current smokers was still observed when controlling for these factors (Additional file 1: Figure S2).

Current smokers were also observed to have significantly lower diversity when the analysis was limited to the FD/ID cohort (Fig. 2c, d). However, this was not the case for CD (Fig. 2e, f). While factors including time since diagnosis and previous surgery in the CD cohort did not impact diversity, patients treated with monoclonal antibody therapies (anti-TNF or anti-integrin) had greater MAM diversity (Additional file 1: Figure S3). Controlling for monoclonal antibody therapy in the CD cohort did not alter the findings regarding diversity and smoking status (Additional file 1: Figure S3).

### Small intestinal MAM composition is altered in smokers

**Fig. 3:**
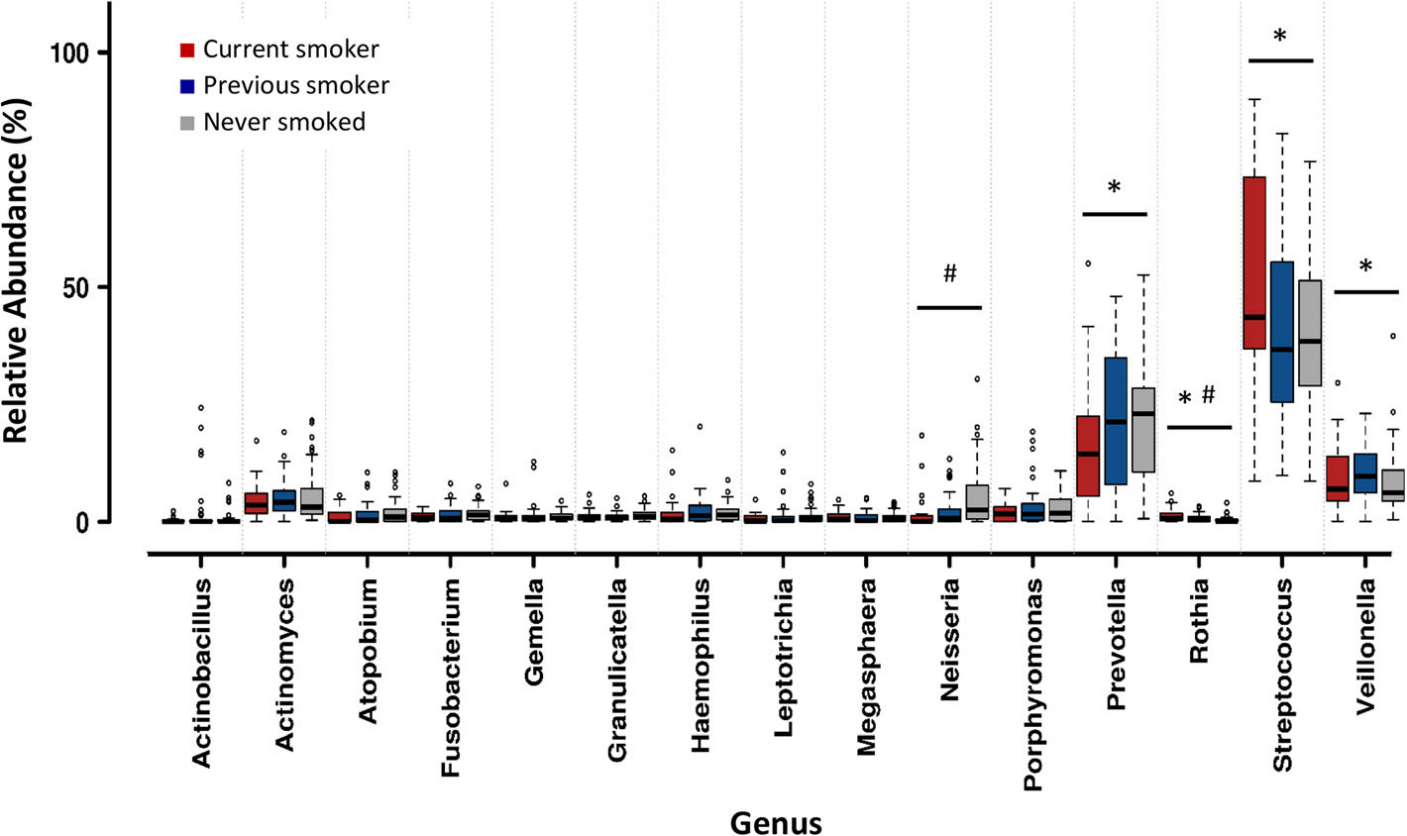
Relative abundances of bacterial genera present in the duodenal (2nd part) mucosa-associated microbiota of all patients. Patients were grouped based on smoking status. Data was normalised via total sum scaling and is expressed as relative abundance. The 15 most abundant genera are displayed. Error bars represent standard deviation. * *p* < 0.005, FDR *q* < 0.05, Kruskal-Wallis with false discovery rate (FDR); # *p* < 0.05 ALDEx2 Wilcoxon rank test with Benjamini-Hochberg (BH) correction. o—order

At the phylum level, the relative abundance of *Firmicutes* was significantly greater in current smokers compared to never smokers, while *Bacteroidetes* and *Actinobacteria* were significantly lower in current smokers (KW *p* < 0.005, FDR *q* < 0.01) (Additional file 1: Table S4). At the genus level, the relative abundances of *Streptococcus*, *Rothia*, and *Veillonella* were greater in current smokers compared to those who had never smoked (KW *p* < 0.005, FDR *q* < 0.05), whereas the relative abundance of *Prevotella* was significantly lower in current smokers (KW *p* < 0.0005, FDR *q* < 0.05) (Fig. 3, Additional file 1: Table S5). Using parallel analyses on raw read data using ALDEx2 (for analysis of compositional data; Additional file 1: S4 and S5), the significant differences in the phyla *Firmicutes* and *Actinobacteria*, along with the genus *Rothia*, between current and never smokers were confirmed. In addition, a significant difference in the genus *Neisseria* was identified, with this taxon being lower in relative abundance in current smokers.

A multivariate analysis was performed to account for patient age, sex, BMI, PPI use, and diagnosis (FD/ID/CD). None of these factors resulted in differences in any phyla or genera in the model (Additional file 1: Tables S6 and S7). In addition, this model confirmed the results in regard to smoking status, for *Firmicutes*, *Bacteroidetes,* and *Actinobacteria* at the phylum level (Additional file 1: Table S6), and *Streptococcus*, *Veillonella*, and *Prevotella* at the genus level (Additional file 1: Table S7).

At the OTU level, four OTUs affiliated with the genus *Streptococcus*, along with a single *Rothia* sp*.* and a single *Veillonella sp.*, were identified as being significantly different in relative abundance in current smokers compared to never smokers (KW *p* < 0.005, FDR *q* < 0.05) (Additional file 1: Table S8). The differences in three of the *Streptococcus* OTUs were confirmed on multivariate analysis controlling for other patient factors; none of the OTUs were significantly different in abundance using ALDEx2 (Additional file 1: Table S8).

Interestingly, when comparing current to previous smokers, or previous to never smokers, no significant differences at the phylum or genus level of classification were observed (Additional file 1: Tables S4 and S5). At the OTU level, no significant differences between current and previous smokers were observed; however, several *Streptococcus*-, *Veillonella*-, and *Prevotella*-affiliated OTUs showed significantly different relative abundances between previous and never smokers (Additional file 1: Table S8). There were no remarkable effects on these results when CD patients were excluded from the analysis, and no significant differences were observed in the CD group alone (Additional file 1: Table S9).

### Differentiation of smokers based on MAM profiles

**Fig. 4:**
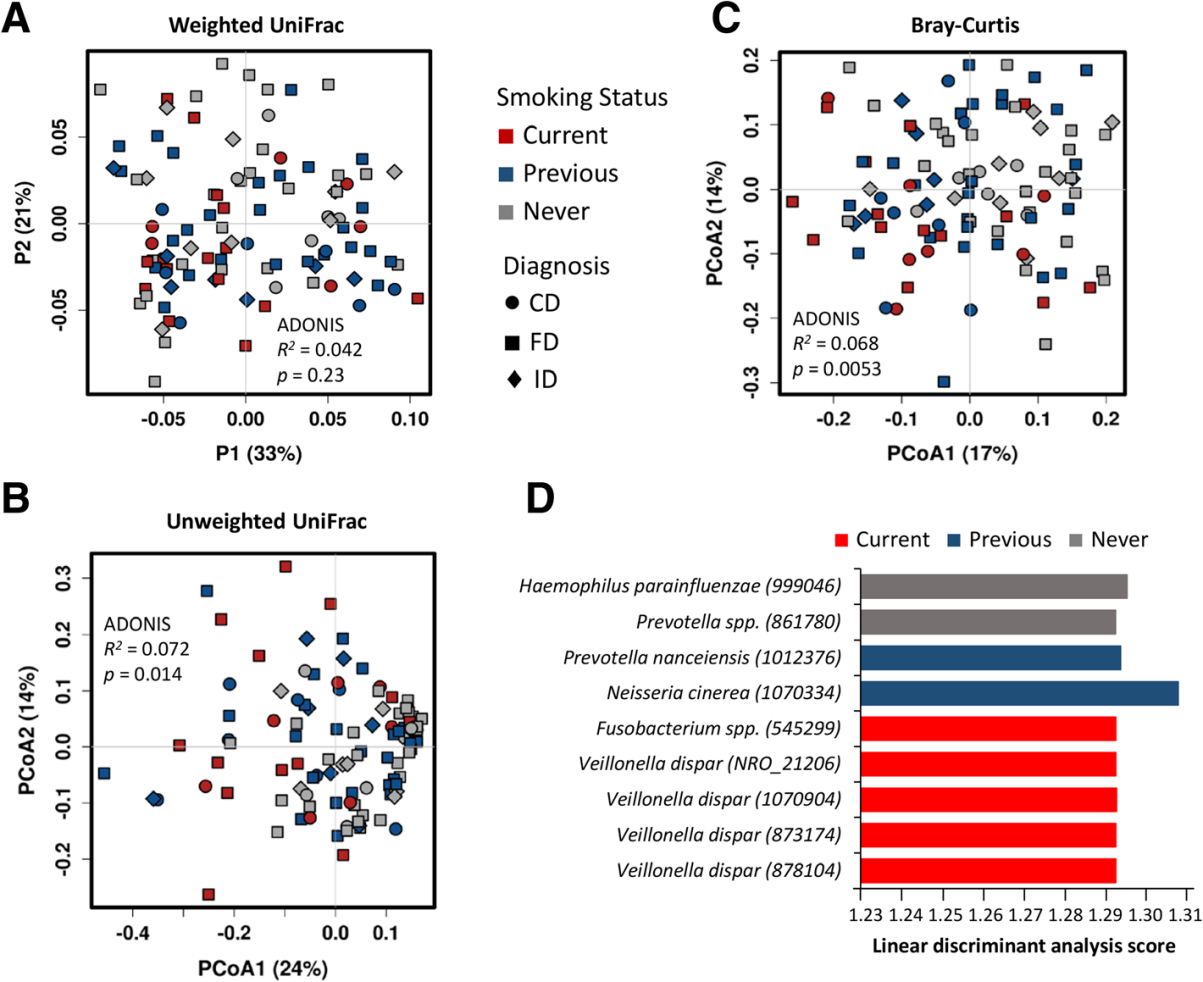
Multivariate analyses to identify bacterial groups that differentiate current smokers from those patients who have never smoked, based on duodenal (2nd part) mucosa-associated microbiota profiles. **a–c** Principal coordinates analysis performed on a weighted UniFrac (**a**), unweighted UniFrac (**b**) and Bray-Curtis (**c**) distance matrices. Each point represents an individual patient, colour coded by smoking status. An assessment of the variation between or “clustering” of samples based on smoking status was assessed via ADONIS, controlling for patient age, sex, BMI, PPI use and diagnosis. Data normalised by random subsampling to even depth. **d** Linear discriminant analysis effect size (LEfSe) method to identify bacterial OTUs that are associated with smoking status. The bar chart represents the strength of contribution of a particular OTU between current, previous and never smokers. Data was normalised via total sum scaling and centred-log ratio transformation. CD—Crohn’s disease; FD—functional dyspepsia; ID—iron deficiency

Principal coordinates analysis of overall MAM profiles suggested some clustering by smoking status (unweighted UniFrac and Bray-Curtis distance matrices), after controlling for sex, age, BMI, PPI use, and diagnosis, although this was not observed in the case of the weighted UniFrac matrix (Fig. 4a–c). No clustering by patient diagnosis (FD/ID/CD) was observed (ADONIS *R*^*2*^ < 0.04, *p* > 0.3).

**Fig. 5:**
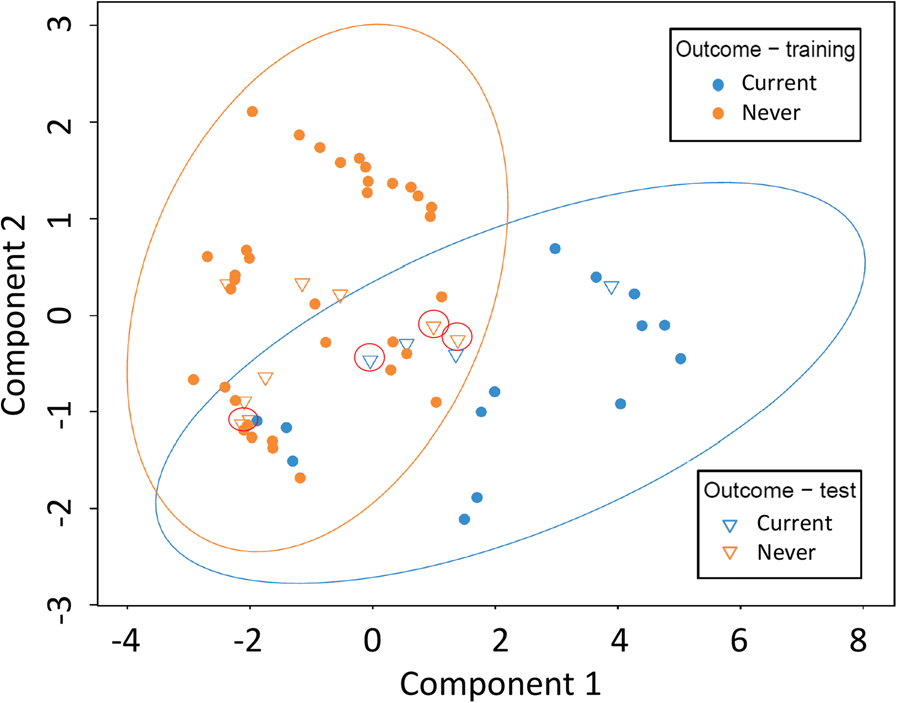
Constrained multivariate analysis using MixMC method (sparse partial least squares discriminant analysis) to differentiate patients based on smoking status (current or never) with respect to duodenal (2nd part) mucosa-associated microbiota profiles. Each point represents an individual patient, colour coded by smoking status. A “training set” which consisted of the FD and ID cohort was used to develop a discriminating model, which was then tested on the CD data set. The figure displays the resulting predicted classification of each sample, with closed circles representing the FD-ID cohort, and open triangles representing the CD cohort. Confidence ellipses (95%) are shown. Samples misclassified by the model are circled in red

Specific taxa contributing to small intestinal MAM profiles, based on smoking status, were revealed by linear discriminant analysis effect size (LEfSe, Fig. 4d). OTUs affiliated with the genera *Veillonella* were discriminatory for current smokers. Particular members of the genus *Prevotella* were also discriminatory for previous smokers and persons never having smoked. To further delineate these differences between bacterial profiles of current and never smokers, a constrained multivariate model was established, using the MAM data from the FD/ID cohort. Taxa affiliated with the *Neisseria*, *Streptococcus*, *Prevotella*, and *Veillonella* genera were identified as the key contributing factors that differentiate current smokers from those who have never smoked (Table 2). The model was validated using the leave-one-out method and was able to correctly classify all never smokers and 13 of 14 current smokers (Table 3, Fig. 5). When the model was applied to the CD patients, however, the performance was not as robust (Table 3).

**Table 2:** Operational taxonomic units (OTUs) contributing to the model discriminating patients based on smoking status

Contributing OTU		Average relative abundance^a^	
Phylum	OTU^b^	Current smokers	Never smokers
*Firmicutes*	*Veillonella dispar* (937248)	6.61	6.00
*Bacteroidetes*	*Prevotella* sp*.* (2222)	0.33	1.58
*Firmicutes*	*Streptococcus* sp*.* (1098340)	30.57	23.02
*Firmicutes*	*Streptococcus* sp*.* (1088134)	4.25	3.43
*Firmicutes*	*Streptococcus* sp*.* (1092300)	0.43	0.35
*Firmicutes*	*Streptococcus* sp*.* (1097208)	0.37	0.28
*Proteobacteria*	*Neisseria* sp*.* (1060621)	0.19	0.6
*Proteobacteria*	*Aggregatibacter* sp*.* (963216)	0.16	0.15
*Bacteroidetes*	*Prevotella intermedia* (72112)	0.48	0.29
*Proteobacteria*	*Neisseria* sp*.* (1092944)	1.72	2.96
*Bacteroidetes*	*Prevotella* sp*.* (851822)	0.72	1.00
*Firmicutes*	*Veillonella* sp*.* (511378)	0.76	0.33
*Proteobacteria*	*Neisseria cinerea* (1070334)	0.088	1.27
*Firmicutes*	*Veillonella dispar* (NRO_21206)	0.11	0.18
*Firmicutes*	*Veillonella dispar* (1019878)	0.63	0.61
*Fusobacteria*	*Fusobacterium* sp*.* (809380)	0.2	0.27
*Proteobacteria*	*Haemophilus parainfluenzae* (832223)	0.47	0.33
*Proteobacteria*	*Haemophilus parainfluenzae* (920105)	0.77	0.71
*Bacteroidetes*	*Prevotella tannerae* (38227)	0.71	0.23
*Firmicutes*	*Oribacterium sp.* (527630)	0.19	0.52

^a^The average relative abundance (%) for each OTU is shown (for the FD-ID training set; data is not transformed). ^b^ The number in brackets refers to the Greengenes OTU reference number; NRO—new reference OTU)

**Table 3:** Classification efficiency of the MixOmics model

Training set (leave-one-out validation) FD and ID cohort			Test set CD cohort		
	Correctly classified (%)	Incorrectly classified (%)		Correctly classified (%)	Incorrectly classified (%)
Current smoker	93	7	Current smoker	43	57
Never smoker	100	0	Never smoker	83	17

Efficiency of the mixOmics model (sparse partial least squares discriminant analysis) in differentiating patients based on smoking status (current or never) with respect to bacterial profiles. A “training set” which consisted of the FD and ID cohort was used to develop a discriminating model, which was validated using the ‘leave-one-out’ method. The model was then tested on the CD data set

In summation, there are hallmark upper small intestinal MAM profiles that differentiate between active smokers and persons who have never smoked. For those persons categorised as previous smokers, there is a partial but perhaps not complete restoration of the MAM.

## Discussion

This is the first study that has assessed the effects of cigarette smoking on the small intestinal MAM and highlights the importance of considering smoking as a factor in clinical studies of the microbiota. Our data reveal that cigarette smoking, both current and previous, alters the bacterial community and reduces diversity, both, to the best of our knowledge, novel observations regarding the upper small intestinal MAM. These changes likely translate into functional differences at the host-microbe interface, which may be relevant to the risk and clinical course of inflammatory conditions affecting the intestine. This is of particular relevance given the large body of work that indicates smoking is a risk factor for important GI diseases, including inflammatory bowel disease, irritable bowel syndrome, *Clostridium difficile* infection, and duodenal ulcer [1–3, 46], many of which have also been associated with alterations of the microbiota.

For individuals who had never smoked, small intestinal MAM diversity was significantly greater than for current smokers. A number of hypotheses relating to this finding, along with the observed alterations to the composition of the bacterial community, could be suggested based on the known effects of smoking, including alterations to the immune system, direct antimicrobial activity [17], and changes to oxygen tension [16]. Overall, a diverse microbiota is generally associated with health, and alterations to immune homeostasis, along with a reduction in diversity induced through smoking, could be suggested to contribute to the adverse impact of smoking on the disease states in which microbiota-immune interactions are considered important. Further studies specifically investigating these interactions are warranted.

We included previous smokers in our analyses, and trends indicated that the previous smokers group may represent an intermediate between smokers and those who have never smoked. In particular, previous smokers had a reduced diversity of the MAM compared to individuals who had never smoked. It has been suggested that quitting may induce a relatively rapid return to a “healthy” microbiota in the stool [13] and oral cavity [12]. However, given patients can tend to under-report their smoking habits [47], it may be speculated that some patients in the previous smokers cohort may represent current smokers under-reporting their status. Time since ceasing smoking, as well as other factors such as environmental tobacco exposure (passive smoking), may also influence the results, and thus, further investigation of these factors would provide more specific insight into the beneficial effects of quitting smoking on the microbiota and associated disease risks.

Smoking did not alter the total density of bacteria present on the small intestinal mucosa. This suggests that the reduced diversity observed in current smokers is not the result of overgrowth of certain members of the microbiota, which would result in higher load and lower diversity, nor it is a result of an overall decrease in bacteria adherent to the mucosa. Rather, our results indicate a shift in composition within the existing microbial community. A particular impact on the small intestinal MAM from smoking is the reduction of the relative abundance *Prevotella* and *Neisseria* spp*.* and an increased relative abundance of *Firmicutes*, principally *Streptococcus* spp*.*, and *Veillonella spp.*, along with the genus *Rothia* (*Actinobacteria*)*,* in current smokers, compared to those persons who have never smoked. These differentiating taxa were identified by both models used in our study.

Interestingly, a number of these taxa have been identified in studies investigating the effects of smoking on the oral cavity, both culture-based studies [48], and more recently, microbiota profiling studies, which reported increased relative abundances of *Streptococcus* spp*.* and decreased relative abundances of *Neisseria* spp*.* among others [12]. There is a clear overlap between the taxa observed in the oral cavity and the small intestine, particularly at broader taxonomic levels, and the oral microbiota has been suggested as a driver of the composition of the gastric microbiota [49]. It would be informative to consider the impact of oral health on the small intestinal microbiota, both generally and in the context of smoking, for example, using matched saliva and biopsy samples, particularly given the negative impact smoking has on oral health and the risk of caries [50].

A variety of mechanisms may be relevant regarding the influence of smoking on particular members of the microbiota. Oxygen tension has been suggested as an important driver of changes, with microaerophilic and fermentative (anaerobic) bacteria able to predominate due to lower oxygenation [11, 51]. The differences we observe in *Neisseria, Streptococcus*, and *Rothia* spp. in current smokers indicate that changes in oxygen tension in the small intestine may be a strong selective pressure on the MAM, but there are also likely to be other physicochemical factors in play. For instance, the relative abundance of select *Prevotella*- and *Veillonella*-affiliated OTUs were discriminatory of persons based on smoking status. These bacteria are strict anaerobes and likely to be sensitive to the oxygen radicals produced as a consequence of smoking [52, 53]. Furthermore, alterations in duodenal bicarbonate secretion [19] and lower duodenal pH [18] in smokers also provide selective pressure, with particular impact on the growth of *Neisseria* that is much more sensitive to acid conditions [54], whereas *Streptococcus* and *Rothia* spp. are acidogenic and acid tolerant.

A number of studies have recently associated alterations in the small intestinal MAM with various disease states. A particular focus has been Coeliac disease, with alterations to the microbiota, including lower diversity, observed in adult patients with untreated disease or those with disease refractory to treatment [22, 23]. Interestingly, in a cohort of patients with type 1 diabetes, a reduction in relative abundance of *Proteobacteria* present in the small intestinal MAM was observed, although this study did not differentiate between children and adults [24]. In chronic liver disease, again, changes in the relative abundance of taxa affiliated with the *Proteobacteria* and *Firmicutes* phyla were observed [25]. However, none of these studies controlled for, or indeed reported, smoking status. Our study indicates smoking as a relevant confounding factor that may preclude or confound identification of disease-specific microbial changes if not considered.

A major strength of our study was the collection of intestinal biopsy samples using biopsy forceps designed to sample the MAM and preclude contamination from the lumen or other regions of the GI tract/oral cavity during sampling (Brisbane Aseptic Biopsy Device) [29]. Thus, the data generated here can be considered to specifically reflect the upper small intestinal MAM and the smoking related changes particular to this site. As our own data highlights, in addition to sampling methodology, environmental factors also have an important impact on the microbiota, and thus, we have considered a variety of factors including age, sex, BMI, and PPI use. One limitation is that the impact of diet on the small intestinal MAM is very poorly characterised, and we did not have access to dietary history for this patient cohort. However, endoscopic procedures, during which biopsy samples were obtained, were undertaken following overnight fasting for all patients. It is also possible that unique medication combinations that individual patients are exposed to, depending on their medical history, may influence the microbiota and increase variation between individuals.

Our study included a modest number of Crohn’s disease patients (*n* = 20). While there were no substantial differences in the duodenal MAM between the FD/ID cohort and the CD cohort, the observed impacts of smoking were not as pronounced when CD patients were considered alone. This may be related to the clinical history of patients or their immune status; however, even though treatment with monoclonal antibody therapy (anti-TNF/anti-integrin) resulted in higher MAM diversity, this did not explain the differing impact of smoking on CD patients compared to the rest of the cohort (FD/ID). The results may also be partly driven by the small sample size for CD patients. It would be informative to undertake these analyses on a larger group of CD patients, with the addition of biopsies from sites relevant to specific inflammation/lesion patterns in individuals, given the well-documented risk associated with smoking in this disorder.

## Conclusions

In summary, this study provides important new insights into the impact of cigarette smoking on the MAM. The reduction in diversity, along with particular bacterial taxa, may have implications for GI disorders in which the microbiota is also implicated. Studies investigating the MAM, particularly in the small intestine, must consider smoking status of participants, as this represents a potentially significant confounding variable.

## Additional files

Additional file 1: **Figure S1.** Bacterial load on the small intestinal mucosa in all patients. **Figure S2.** Bacterial diversity (Chao1 index) within the mucosa-associated microbiota from duodenal biopsies from patients classified as having a functional dyspepsia (FD), iron deficiency (ID), or Crohn’s disease (CD). **Figure S3.** Bacterial diversity (Chao1 index) within the mucosa-associated microbiota from duodenal biopsies from patients with Crohn’s disease (CD). **Table S1.** Additional characteristics for the functional/iron deficiency patients included in the cohort (*n* = 82). **Table S2.** Additional characteristics for the 20 CD patients included in the cohort, including CD medications. **Table S3.** OTU table with raw read counts used for subsequent data analysis. **Table S4.** Relative abundances of bacterial phyla present in the duodenal mucosal microbiota of all patients. **Table S5.** Relative abundances of bacterial genera present in the duodenal mucosal microbiota of all patients. **Table S6.** Bacterial phyla present in the duodenal mucosal microbiota of all patients. **Table S7.** Bacterial genera present in the duodenal mucosal microbiota of all patients. **Table S8.** Bacterial OTUs that were observed to have significantly different relative abundances in the duodenal mucosal microbiota of all patients. **Table S9.** *p* values comparing relative abundances of bacterial genera present in the duodenal mucosal microbiota of either functional dyspepsia (FD) and iron deficiency (ID) patients (Table A), or Crohn’s disease (CD) patients (Table B). Patients were grouped based on smoking status (current smoker, previous smoker, or having never smoked). (PDF 822 kb)

Additional file 2: Supplementary Methods. (PDF 163 kb)

Additional file 3: Supplementary Table S3 - OTU table with raw read counts. (XLSX 46 kb)

## Abbreviations

BMI: Body mass index
CD: Crohn’s disease
FD: Functional dyspepsia
FDR: False discovery rate
gDNA: Genomic DNA
GI: Gastrointestinal
ID: Iron deficiency
KW: Kruskal-Wallis
MAM: Mucosa-associated microbiota
OTUs: Operational taxonomic units
PPI: Proton pump inhibitor

## References

[1] To N, Gracie DJ, Ford AC (2016). Systematic review with meta-analysis: the adverse effects of tobacco smoking on the natural history of Crohn’s disease. Aliment Pharmacol Ther.

[2] Nam SY, Kim BC, Ryu KH, Park BJ (2010). Prevalence and risk factors of irritable bowel syndrome in healthy screenee undergoing colonoscopy and laboratory tests. J Neurogastroenterol Motil.

[3] Kato I, Nomura AM, Stemmermann GN, Chyou PH (1992). A prospective study of gastric and duodenal ulcer and its relation to smoking, alcohol, and diet. Am J Epidemiol.

[4] Botteri E, Iodice S, Bagnardi V, Raimondi S, Lowenfels AB, Maisonneuve P (2008). Smoking and colorectal cancer: a meta-analysis. JAMA.

[5] Pan SY, Morrison H (2011). Epidemiology of cancer of the small intestine. World J Gastrointest Oncol.

[6] Andrews JM, Mountifield RE, Van Langenberg DR, Bampton PA, Holtmann GJ (2010). Un-promoted issues in inflammatory bowel disease: opportunities to optimize care. Intern Med J.

[7] Parasher G, Eastwood GL (2000). Smoking and peptic ulcer in the Helicobacter pylori era. Eur J Gastroenterol Hepatol.

[8] Kiely CJ, Pavli P, O'Brien CL. The role of inflammation in temporal shifts in the inflammatory bowel disease mucosal microbiome. Gut Microbes. 2018;1–25. 10.1080/19490976.2018.1448742. 10.1080/19490976.2018.1448742 PMC6287691 29543557

[9] Coker OO, Dai Z, Nie Y, Zhao G, Cao L, Nakatsu G, Wu WK, Wong SH, Chen Z, Sung JJY, et al. Mucosal microbiome dysbiosis in gastric carcinogenesis. Gut. 2018;67(6):1024–32. 10.1136/gutjnl-2017-314281 PMC5969346 28765474

[10] Nakatsu G, Li X, Zhou H, Sheng J, Wong SH, Wu WK, Ng SC, Tsoi H, Dong Y, Zhang N (2015). Gut mucosal microbiome across stages of colorectal carcinogenesis. Nat Commun.

[11] Mason MR, Preshaw PM, Nagaraja HN, Dabdoub SM, Rahman A, Kumar PS (2015). The subgingival microbiome of clinically healthy current and never smokers. ISME J.

[12] Wu J, Peters BA, Dominianni C, Zhang Y, Pei Z, Yang L, Ma Y, Purdue MP, Jacobs EJ, Gapstur SM (2016). Cigarette smoking and the oral microbiome in a large study of American adults. ISME J.

[13] Biedermann L, Zeitz J, Mwinyi J, Sutter-Minder E, Rehman A, Ott SJ, Steurer-Stey C, Frei A, Frei P, Scharl M (2013). Smoking cessation induces profound changes in the composition of the intestinal microbiota in humans. PLoS One.

[14] Verschuere S, Bracke KR, Demoor T, Plantinga M, Verbrugghe P, Ferdinande L, Lambrecht BN, Brusselle GG, Cuvelier CA (2011). Cigarette smoking alters epithelial apoptosis and immune composition in murine GALT. Lab Investig.

[15] Allais L, Kerckhof FM, Verschuere S, Bracke KR, De Smet R, Laukens D, Van den Abbeele P, De Vos M, Boon N, Brusselle GG (2016). Chronic cigarette smoke exposure induces microbial and inflammatory shifts and mucin changes in the murine gut. Environ Microbiol.

[16] Jensen JA, Goodson WH, Hopf HW, Hunt TK (1991). Cigarette smoking decreases tissue oxygen. Arch Surg.

[17] Pavia CS, Pierre A, Nowakowski J (2000). Antimicrobial activity of nicotine against a spectrum of bacterial and fungal pathogens. J Med Microbiol.

[18] Murthy SN, Dinoso VP, Clearfield HR, Chey WY (1978). Serial pH changes in the duodenal bulb during smoking. Gastroenterology.

[19] Ainsworth MA, Hogan DL, Koss MA, Isenberg JI (1993). Cigarette smoking inhibits acid-stimulated duodenal mucosal bicarbonate secretion. Ann Intern Med.

[20] Miller G, Palmer KR, Smith B, Ferrington C, Merrick MV (1989). Smoking delays gastric emptying of solids. Gut.

[21] Moran C, Sheehan D, Shanahan F (2015). The small bowel microbiota. Curr Opin Gastroenterol.

[22] Wacklin P, Laurikka P, Lindfors K, Collin P, Salmi T, Lahdeaho ML, Saavalainen P, Maki M, Matto J, Kurppa K (2014). Altered duodenal microbiota composition in celiac disease patients suffering from persistent symptoms on a long-term gluten-free diet. Am J Gastroenterol.

[23] Nistal E, Caminero A, Herran AR, Perez-Andres J, Vivas S, Ruiz de Morales JM, Saenz de Miera LE, Casqueiro J (2016). Study of duodenal bacterial communities by 16S rRNA gene analysis in adults with active celiac disease vs non-celiac disease controls. J Appl Microbiol.

[24] Pellegrini S, Sordi V, Bolla AM, Saita D, Ferrarese R, Canducci F, Clementi M, Invernizzi F, Mariani A, Bonfanti R (2017). Duodenal mucosa of patients with type 1 diabetes shows distinctive inflammatory profile and microbiota. J Clin Endocrinol Metab.

[25] Chen Y, Ji F, Guo J, Shi D, Fang D, Li L (2016). Dysbiosis of small intestinal microbiota in liver cirrhosis and its association with etiology. Sci Rep.

[26] Kerckhoffs AP, Samsom M, van der Rest ME, de Vogel J, Knol J, Ben-Amor K, Akkermans LM (2009). Lower Bifidobacteria counts in both duodenal mucosa-associated and fecal microbiota in irritable bowel syndrome patients. World J Gastroenterol: WJG.

[27] Zhong L, Shanahan ER, Raj A, Koloski NA, Fletcher L, Morrison M, Walker MM, Talley NJ, Holtmann G (2017). Dyspepsia and the microbiome: time to focus on the small intestine. Gut.

[28] Stanghellini V, Chan FK, Hasler WL, Malagelada JR, Suzuki H, Tack J, Talley NJ (2016). Gastroduodenal disorders. Gastroenterology.

[29] Shanahan ER, Zhong L, Talley NJ, Morrison M, Holtmann G (2016). Characterisation of the gastrointestinal mucosa-associated microbiota: a novel technique to prevent cross-contamination during endoscopic procedures. Aliment Pharmacol Ther.

[30] O Cuiv P, Aguirre de Carcer D, Jones M, Klaassens ES, Worthley DL, Whitehall VL, Kang S, McSweeney CS, Leggett BA, Morrison M: The effects from DNA extraction methods on the evaluation of microbial diversity associated with human colonic tissue. Microb Ecol 2011, 61(2):353–362. 10.1007/s00248-010-9771-x 21153634

[31] Salter SJ, Cox MJ, Turek EM, Calus ST, Cookson WO, Moffatt MF, Turner P, Parkhill J, Loman NJ, Walker AW (2014). Reagent and laboratory contamination can critically impact sequence-based microbiome analyses. BMC Biol.

[32] Caporaso JG, Kuczynski J, Stombaugh J, Bittinger K, Bushman FD, Costello EK, Fierer N, Pena AG, Goodrich JK, Gordon JI (2010). QIIME allows analysis of high-throughput community sequencing data. Nat Methods.

[33] Rideout JR, He Y, Navas-Molina JA, Walters WA, Ursell LK, Gibbons SM, Chase J, McDonald D, Gonzalez A, Robbins-Pianka A (2014). Subsampled open-reference clustering creates consistent, comprehensive OTU definitions and scales to billions of sequences. PeerJ.

[34] DeSantis TZ, Hugenholtz P, Larsen N, Rojas M, Brodie EL, Keller K, Huber T, Dalevi D, Hu P, Andersen GL (2006). Greengenes, a chimera-checked 16S rRNA gene database and workbench compatible with ARB. Appl Environ Microbiol.

[35] Haas BJ, Gevers D, Earl AM, Feldgarden M, Ward DV, Giannoukos G, Ciulla D, Tabbaa D, Highlander SK, Sodergren E (2011). Chimeric 16S rRNA sequence formation and detection in Sanger and 454-pyrosequenced PCR amplicons. Genome Res.

[36] Chao A (1984). Nonparametric-estimation of the number of classes in a population. Scand J Stat.

[37] Faith DP (1992). Conservation evaluation and phylogenetic diversity. Biol Conserv.

[38] Gloor GB, Macklaim JM, Pawlowsky-Glahn V, Egozcue JJ (2017). Microbiome datasets are compositional: and this is not optional. Front Microbiol.

[39] McMurdie PJ, Holmes S (2014). Waste not, want not: why rarefying microbiome data is inadmissible. PLoS Comput Biol.

[40] Fernandes AD, Reid JN, Macklaim JM, McMurrough TA, Edgell DR, Gloor GB (2014). Unifying the analysis of high-throughput sequencing datasets: characterizing RNA-seq, 16S rRNA gene sequencing and selective growth experiments by compositional data analysis. Microbiome.

[41] Zakrzewski M, Proietti C, Ellis JJ, Hasan S, Brion MJ, Berger B, Krause L (2017). Calypso: a user-friendly web-server for mining and visualizing microbiome-environment interactions. Bioinformatics.

[42] Weiss S, Xu ZZ, Peddada S, Amir A, Bittinger K, Gonzalez A, Lozupone C, Zaneveld JR, Vazquez-Baeza Y, Birmingham A (2017). Normalization and microbial differential abundance strategies depend upon data characteristics. Microbiome.

[43] McArdle BH, Anderson MJ (2001). Fitting multivariate models to community data: a comment on distance-based redundancy analysis. Ecology.

[44] Segata N, Izard J, Waldron L, Gevers D, Miropolsky L, Garrett WS, Huttenhower C (2011). Metagenomic biomarker discovery and explanation. Genome Biol.

[45] Le Cao KA, Costello ME, Lakis VA, Bartolo F, Chua XY, Brazeilles R, Rondeau P (2016). MixMC: a multivariate statistical framework to gain insight into microbial communities. PLoS One.

[46] Rogers MA, Greene MT, Saint S, Chenoweth CE, Malani PN, Trivedi I, Aronoff DM (2012). Higher rates of Clostridium difficile infection among smokers. PLoS One.

[47] Connor Gorber S, Schofield-Hurwitz S, Hardt J, Levasseur G, Tremblay M (2009). The accuracy of self-reported smoking: a systematic review of the relationship between self-reported and cotinine-assessed smoking status. Nicotine Tob Res.

[48] Colman G, Beighton D, Chalk AJ, Wake S (1976). Cigarette smoking and the microbial flora of the mouth. Aust Dent J.

[49] Bassis CM, Erb-Downward JR, Dickson RP, Freeman CM, Schmidt TM, Young VB, Beck JM, Curtis JL, Huffnagle GB (2015). Analysis of the upper respiratory tract microbiotas as the source of the lung and gastric microbiotas in healthy individuals. MBio.

[50] Bergstrom J, Eliasson S, Dock J (2000). Exposure to tobacco smoking and periodontal health. J Clin Periodontol.

[51] Ganesan SM, Joshi V, Fellows M, Dabdoub SM, Nagaraja HN, O'Donnell B, Deshpande NR, Kumar PS. A tale of two risks: smoking, diabetes and the subgingival microbiome. ISME J. 2017;11(9):2075–89. 10.1038/ismej.2017.73 PMC5563960 28534880

[52] Tsuchiya M, Asada A, Kasahara E, Sato EF, Shindo M, Inoue M (2002). Smoking a single cigarette rapidly reduces combined concentrations of nitrate and nitrite and concentrations of antioxidants in plasma. Circulation.

[53] Haj Mouhamed D, Ezzaher A, Neffati F, Douki W, Gaha L, Najjar MF (2011). Effect of cigarette smoking on plasma uric acid concentrations. Environ Health Prev Med.

[54] Svensater G, Larsson UB, Greif EC, Cvitkovitch DG, Hamilton IR (1997). Acid tolerance response and survival by oral bacteria. Oral Microbiol Immunol.

