# Supplementary material for: Influence of cigarette smoking on the human duodenal mucosa-associated microbiota
Source: Microbiome. 2018 Aug 29;6:150. doi: 10.1186/s40168-018-0531-3 (PMC6116507; doi:10.1186/s40168-018-0531-3)
Supplement: Supplementary file 1 — Figure S1. Bacterial load on the small intestinal mucosa in all patients. Figure S2. Bacterial diversity (Chao1 index) within the mucosa-associated microbiota from duodenal biopsies from patients classified as having a functional dyspepsia (FD), iron deficiency (ID), or Crohn’s disease (CD). Figure S3. Bacterial diversity (Chao1 index) within the mucosa-associated microbiota from duodenal biopsies from patients with Crohn’s disease (CD). Table S1. Additional characteristics for the functional/iron deficiency patients included in the cohort (n = 82). Table S2. Additional characteristics for the 20 CD patients included in the cohort, including CD medications. Table S3. OTU table with raw read counts used for subsequent data analysis. Table S4. Relative abundances of bacterial phyla present in the duodenal mucosal microbiota of all patients. Table S5. Relative abundances of bacterial genera present in the duodenal mucosal microbiota of all patients. Table S6. Bacterial phyla present in the duodenal mucosal microbiota of all patients. Table S7. Bacterial genera present in the duodenal mucosal microbiota of all patients. Table S8. Bacterial OTUs that were observed to have significantly different relative abundances in the duodenal mucosal microbiota of all patients. Table S9. p values comparing relative abundances of bacterial genera present in the duodenal mucosal microbiota of either functional dyspepsia (FD) and iron deficiency (ID) patients (Table A), or Crohn’s disease (CD) patients (Table B). Patients were grouped based on smoking status (current smoker, previous smoker, or having never smoked). (PDF 822 kb) [file 40168_2018_531_MOESM1_ESM.pdf]

SUPPLEMENTARY FIGURES AND TABLES

INFLUENCE OF CIGARETTE SMOKING ON THE HUMAN DUODENAL MUCOSA ASSOCIATED MICROBIOTA

Erin R. Shanahan[1,2,3,#], Ayesha Shah[1,2], Natasha Koloski[1,4], Marjorie Walker[4], Nicholas J Talley[4], Mark Morrison[2,3*], and Gerald J Holtmann[1,2*]

1. Department of Gastroenterology and Hepatology, Princess Alexandra Hospital, and Faculty of Medicine, The University of Queensland, Woolloongabba, Queensland, Australia
2. Translational Research Institute, Woolloongabba, Queensland, Australia
3. The University of Queensland Diamantina Institute, Faculty of Medicine, The University of Queensland, St Lucia, Queensland, Australia
4. Faculty of Health and Medicine, University of Newcastle, Newcastle, New South Wales, Australia

# Current Address: School of Life and Environmental Sciences, Charles Perkins Centre, The University of Sydney, Camperdown, New South Wales, Australia

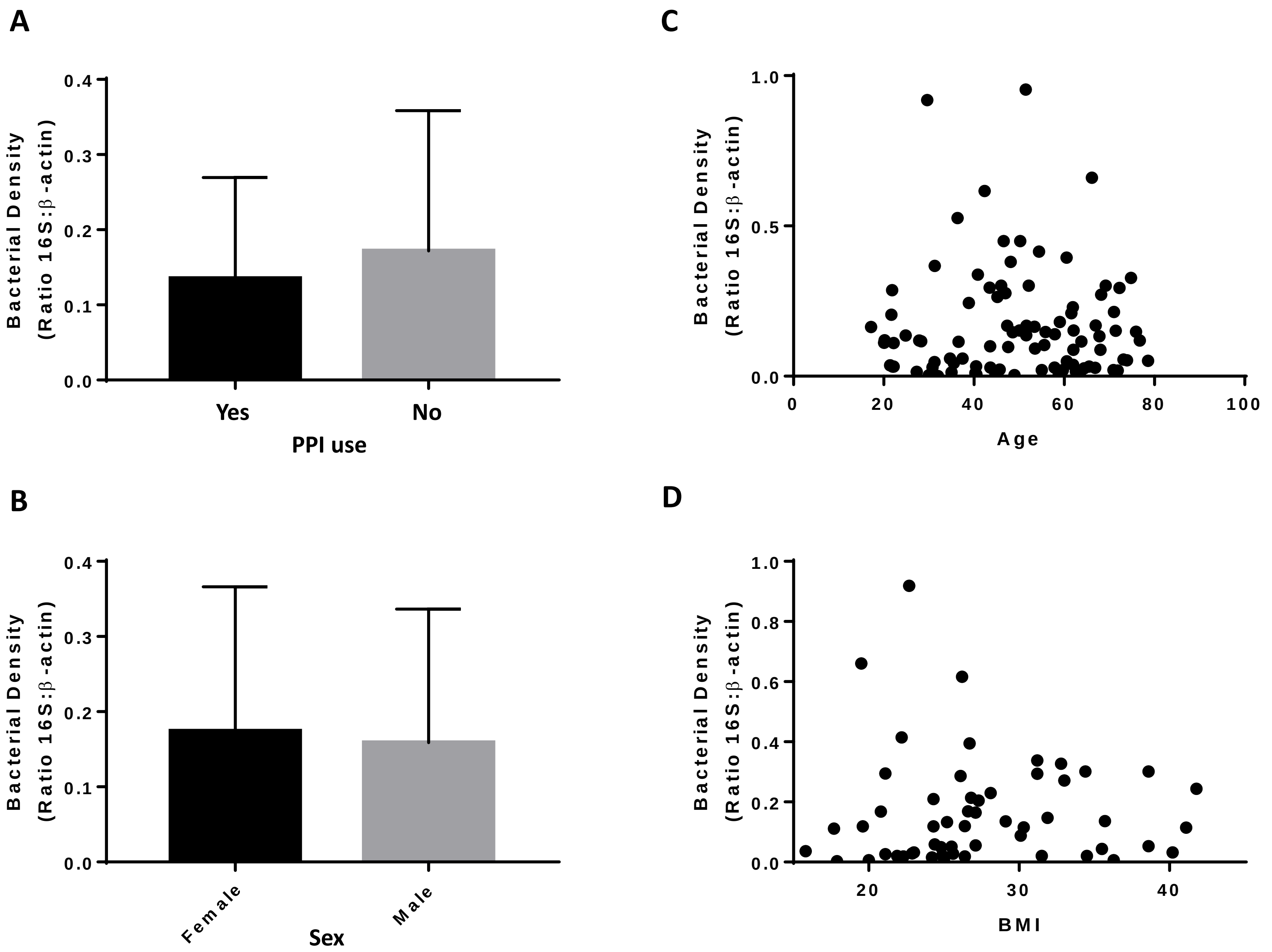

Figure S1. Bacterial load on the small intestinal mucosa in all patients. Load was assessed by qPCR and expressed as the ratio between copies of the bacterial 16S rRNA gene and copies of the human beta-actin gene. Mann-Whitney test (A, B) /Spearman correlations (C, D) were performed as appropriate. **(A)** No significant difference in load was observed based on proton pump inhibitor (PPI) use (p=0.36). **(B)** No significant difference in load was observed based on patient sex (p=0.89). **(C)** No significant correlation between patient age and bacterial load was observed (r=0.04, p=0.72). **(D)** No significant correlation between patient body mass index (BMI) and bacterial load was observed (r=0.17, p=0.22).

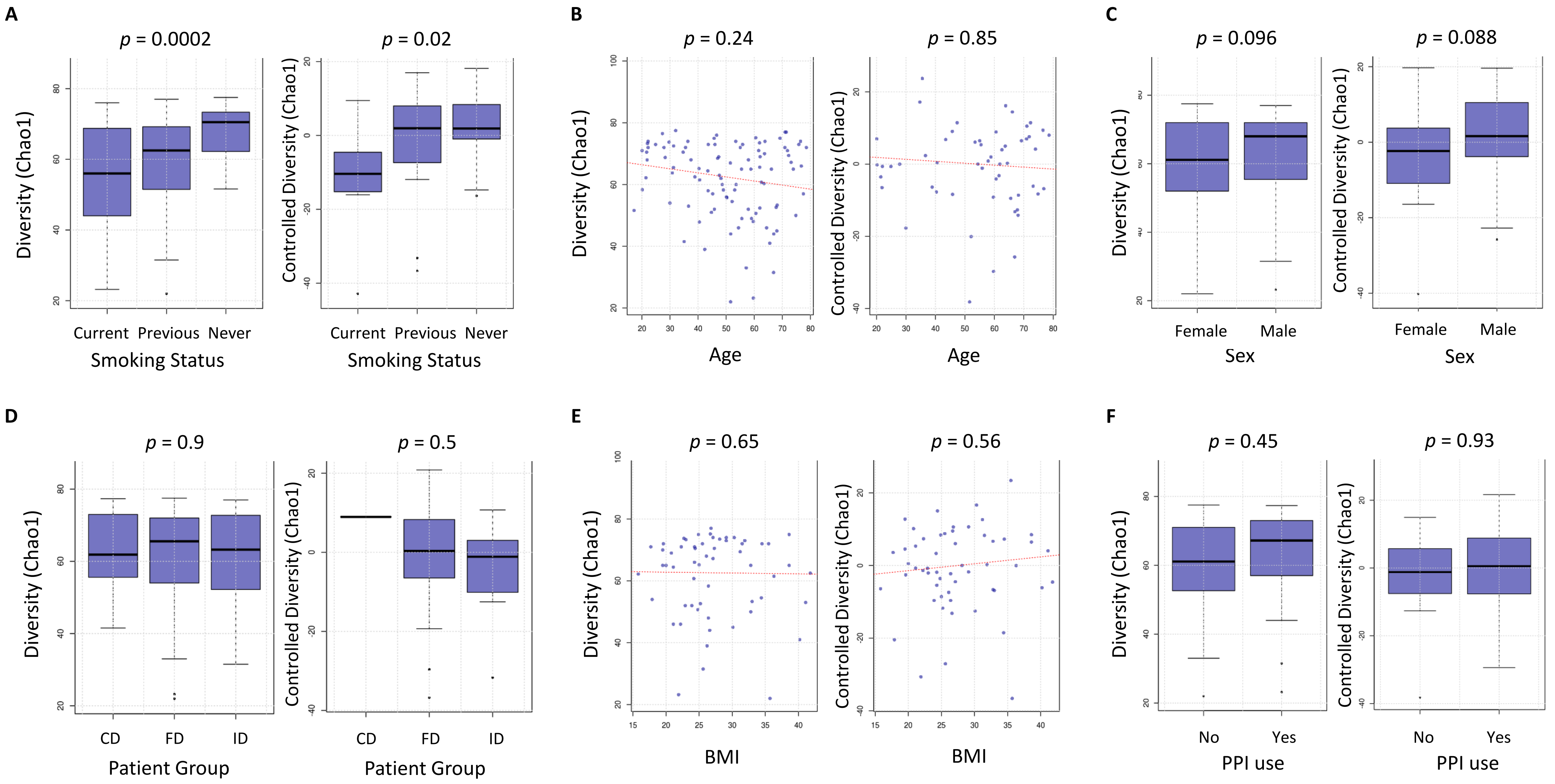

Figure S2. Bacterial diversity (Chao1 index) within the mucosa-associated microbiota from duodenal biopsies from patients classified as having a functional dyspepsia (FD), iron deficiency (ID) or Crohn's disease (CD). A univariate and multivariate analysis (linear regression model) was performed including smoking status **(A)**, age **(B)**, sex **(C)**, patient group **(D)**, body mass index (BMI) **(E)** and proton pump inhibitor (PPI) use **(F)**. Mean and Standard deviation are shown on plots. For univariate analysis, Kruskal-Wallis, Mann-Whitney or Spearman correlation was used as appropriate.

A

Factor	*p*-value
Anti-TNF/Anti-integrin	0.043
ASA - aminosalicylic acid	0.75
AZA - azathioprine; 6-MP - 6-mercaptopurine	0.65
MTX – methotrexate	0.48
Prior Surgeries	0.11
Time since diagnosis	0.3
Montreal Classification	0.45

B

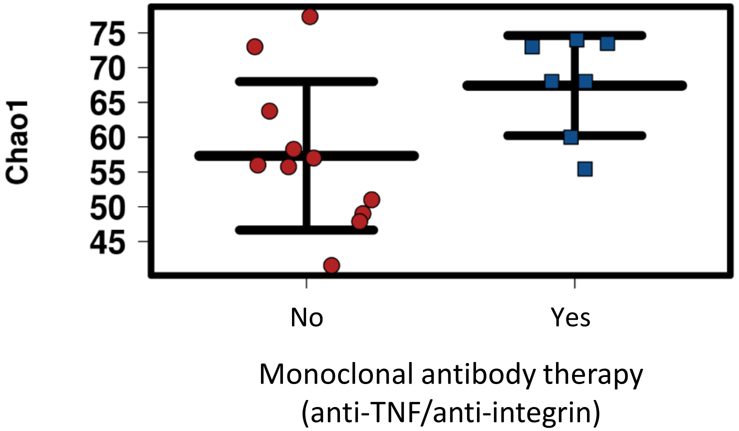

C

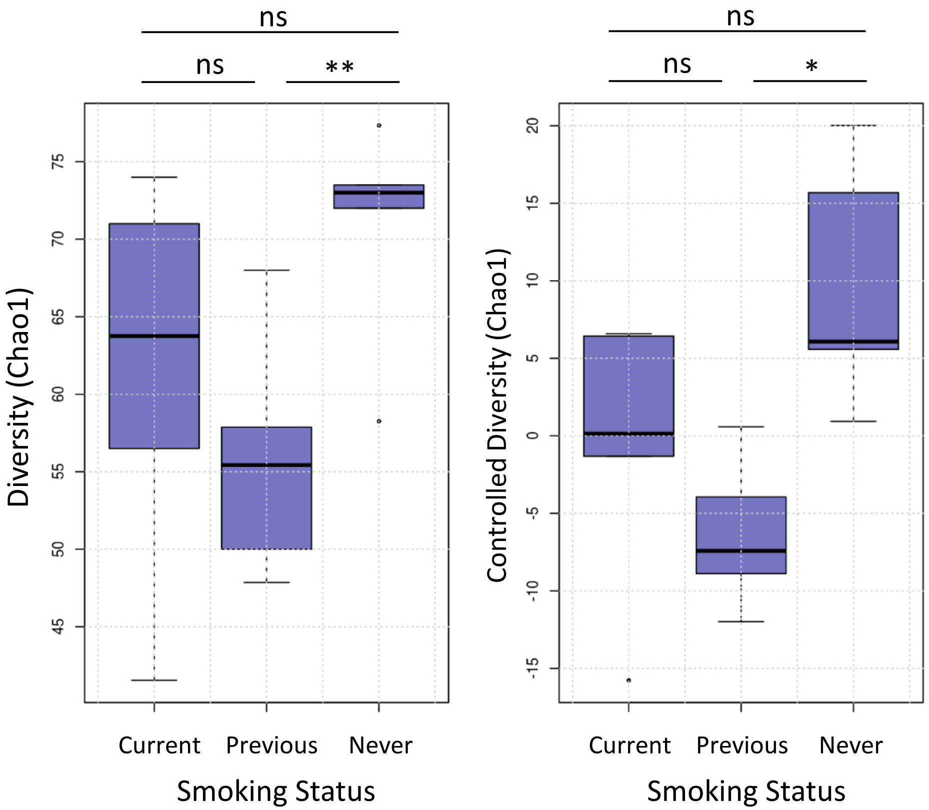

Figure S3. Bacterial diversity (Chao1 index) within the mucosa-associated microbiota from duodenal biopsies from patients with Crohn's disease (CD). **(A)** A univariate analysis (Kruskal-Wallis/Spearman correlation as appropriate) was performed on factors including medication, prior surgery, time since diagnosis and Montreal classification of patients. **(B)** Plot showing the diversity (Chao1 index) of patients based on treatment with monoclonal antibody therapy or other/no treatment. **(C)** Univariate and multivariate analysis (linear regression model) was performed based on smoking status and controlling for treatment with monoclonal antibody therapy.

Table S1. Additional characteristics for the functional/iron deficiency patients included in the cohort (n=82).

	**Number of patients**	**Percentage (%)**
IBS-like symptoms	47	57.3
Gastro-oesophageal reflux	16	19.5
Chronic obstructive pulmonary disease	5	6.1
Asthma	9	11
Diabetes (Type 2)	17	20.7
Kidney disease	6	7.3
Previous cancer (breast/prostate/melanoma)	5	6.1
Post-traumatic stress disorder	4	4.9

Table S2. Additional characteristics for the 20 CD patients included in the cohort, including CD medications.

	**Number of patients**
**Disease Classification**	
Ileal	6
Ileo-colonic	13
Ileo-colonic and upper GI	1[a]
**Medications[b]**	
Anti-TNF/ Anti-integrin	7
AZA - azathioprine; 6-MP - 6-mercaptopurine	6
ASA - aminosalicylic acid	3
MTX – methotrexate	5
None	6
**Previous Surgery**	
None	8
1-2 procedures	11
3-4 procedures	1
**Time since diagnosis**	
≤ 5 years	5
≤ 10 years	2
≥ 11 years	10
Unknown	3

[a] No patients had upper GI involvement with the exception of one patient, however this patient had no evidence of any current lesions/inflammation in the duodenum at endoscopy for this study. [b] Six patients were on dual therapy.

Table S3. (Please see separate excel file). OTU table with raw read counts used for subsequent data analysis.

Table S4. Relative abundances of bacterial phyla present in the duodenal mucosal microbiota of all patients. Patients were grouped based on smoking status (Current smoker, previous smoker, or having never smoked). **(A)** Data were normalised via total sum scaling and centred-log ratio transformation, and expressed as mean relative abundance. Significance was determined utilising Kruskal-Wallis with false discovery rate (FDR) correction for multiple comparisons. **(B)** Non-normalised (raw) data were analysed for differential relative abundance utilising ALDEx2 (Wilcoxon Rank Test with Benjamini-Hochberg (BH) correction; data are expressed as mean relative abundance (non-transformed).

A

Phylum	*p*-value (Current vs Never)	FDR *q*-value (Current vs Never)	*p*-value (Current vs Previous)	FDR *q*-value (Current vs Previous)	*p*-value (Previous vs Never)	FDR *q*-value (Previous vs Never)	Current mean[a]	Previous mean[a]	Never mean[a]
Proteobacteria	0.95	1	0.45	0.68	0.58	1	-1.2	1.44	0.91
TM7	0.99	1	1	1	1	1	-13	-9.5	-7.6
Firmicutes	0.00033	0.0011	0.058	0.17	0.078	0.23	5.7	4.43	3.34
Bacteroidetes	0.0027	0.0054	0.17	0.34	0.059	0.23	4.01	3.29	2.26
Fusobacteria	0.037	0.055	1	1	1	1	0.81	-1.6	0.03
Actinobacteria	0.00037	0.0011	0.025	0.15	0.13	0.26	3.28	1.93	1.05

a These means represent relative abundance (%) data that has been centred-log ratio transformed. Statistical analysis was performed on this transformed data.

B

Phylum	*p*-value (Current vs Never)	BH *p*-value (Current vs Never)	*p*-value (Current vs Previous)	BH *p*-value (Current vs Previous)	*p*-value (Previous vs Never)	BH *p*-value (Previous vs Never)	Current mean[b]	Previous mean[b]	Never mean[b]
Proteobacteria	0.059	0.107	0.125	0.249	0.508	0.537	4.9	7.01	8.37
TM7	0.275	0.342	0.502	0.580	0.231	0.358	0.16	0.17	0.33
Firmicutes	0.0015	0.009	0.070	0.177	0.116	0.319	66.82	57.11	55.22
Bacteroidetes	0.293	0.351	0.737	0.751	0.079	0.305	19.25	25.34	24.81
Fusobacteria	0.390	0.405	0.240	0.349	0.475	0.547	1.88	2.91	3.07
Actinobacteria	0.007	0.021	0.054	0.177	0.371	0.488	6.99	7.46	8.2

b ALDEx2 analysis was performed on raw read count data; The means shown here are relative abundance (%).

Table S5. Relative abundances of bacterial genera present in the duodenal mucosal microbiota of all patients. Patients were grouped based on smoking status (Current smoker, previous smoker, or having never smoked). **(A)** Data were normalised via total sum scaling and centred-log ratio transformation, and expressed as mean relative abundance. Significance was determined utilising Kruskal-Wallis with false discovery rate (FDR) correction for multiple comparisons. **(B)** Non-normalised (raw) data were analysed for differential relative abundance utilising ALDEx2 (Wilcoxon Rank Test with Benjamini-Hochberg (BH) correction); data are expressed as mean relative abundance (non-transformed). (p) – phylum; (c) – class; (o) – order; (f) – family.

A

Genus	*p*-value (Current vs Never)	FDR *q*-value (Current vs Never)	*p*-value (Current vs Previous)	FDR *q*-value (Current vs Previous)	*p*-value (Previous vs Never)	FDR *q*-value (Previous vs Never)	Current mean[a]	Previous mean[a]	Never mean[a]
Actinobacillus	1	1	1	1	1	1	-4	-2.4	-2.7
Actinomyces	0.13	0.36	0.82	1	0.053	0.22	4.68	5.77	4.49
Aggregatibacter	1	1	1	1	1	1	-4.3	-6.2	-6.7
Atopobium	1	1	1	1	1	1	-2.2	-0.87	0.52
***Bacilli* (c)**	1	1	1	1	1	1	-2.2	-2.1	-1.8
Bulleidia	1	1	1	1	1	1	-6.7	-5.3	-3.2
Fusobacterium	0.013	0.065	0.059	0.96	0.82	1	4.43	2	3.04
Gemella	0.09	0.28	0.38	1	0.34	0.94	3.35	2.33	2.43
***Gemellales* (o)**	1	1	1	1	1	1	-1.3	-1.1	0.39
Granulicatella	0.052	0.19	0.23	0.96	0.41	1	4.46	2.54	2.69
Haemophilus	0.5	1	0.67	1	0.07	0.22	1.65	3.75	3.15
Helicobacter	1	1	1	1	1	1	-11	-12	-17

Lachnospiraceae **(f)**	1	1	1	1	1	1	-1.5	-2.5	-0.78
Lactobacillales **(o)**	0.03	0.12	0.46	1	0.049	0.22	4.31	3.65	2.23
Leptotrichia	0.72	1	1	1	1	1	-0.14	-1.3	0.33
Megasphaera	1	1	1	1	1	1	-1.4	-1	-0.75
Neisseria	0.47	1	0.64	1	0.92	1	-0.72	1.42	2.5
Oribacterium	1	1	1	1	1	1	-5.8	-3.6	-0.73
Peptostreptococcus	1	1	1	1	1	1	-7.8	-7	-5
Porphyromonas	0.21	0.52	0.62	1	0.0089	0.069	1.91	3.82	1.83
Prevotella	0.00031	0.0078	0.23	0.96	0.0079	0.069	8.86	7.88	5.94
Rothia	0.0043	0.027	0.15	0.96	0.07	0.22	4.38	1.51	0.46
Streptococcus	0.0015	0.012	0.14	0.96	0.011	0.069	10.38	8.78	6.82
TM7 **(p)**	1	1	1	1	1	1	-7.6	-4.7	-3.7
Veillonella	0.0015	0.012	0.18	0.96	0.0043	0.069	8.26	6.8	5.15

a These means represent relative abundance (%) data that has been centred-log ratio transformed. Statistical analysis was performed on this transformed data.

B

Genus	*p*-value (Current vs Never)	BH *p*-value (Current vs Never)	*p*-value (Current vs Previous)	BH *p*-value (Current vs Previous)	*p*-value (Previous vs Never)	BH *p*-value (Previous vs Never)	Current mean[b]	Previous mean[b]	Never mean[b]
Actinobacillus	0.501	0.769	0.750	0.928	0.464	0.675	0.29	2.15	0.84
Actinomyces	0.713	0.898	0.616	0.923	0.277	0.582	4.35	5.25	5.78
Aggregatibacter	0.109	0.346	0.352	0.885	0.433	0.627	0.16	0.16	0.15
Atopobium	0.479	0.805	0.856	0.941	0.271	0.584	1.26	1.45	1.93

Bacilli **(c)**	0.606	0.836	0.675	0.924	0.587	0.755	0.24	0.24	0.27
Bulleidia	0.518	0.759	0.722	0.929	0.275	0.569	0.24	0.39	0.34
Fusobacterium	0.535	0.836	0.348	0.905	0.538	0.713	1.19	1.55	1.77
Gemella	0.437	0.780	0.440	0.915	0.887	0.922	1.15	1.39	1.09
Gemellales **(o)**	0.763	0.899	0.530	0.917	0.442	0.658	0.27	0.56	0.4
Granulicatella	0.843	0.920	0.735	0.936	0.887	0.914	1.29	1.1	1.42
Haemophilus	0.340	0.716	0.297	0.899	0.718	0.807	2.24	2.36	1.97
Helicobacter	0.041	0.159	0.357	0.901	0.056	0.223	0.051	0.0084	0.027
Lachnospiraceae **(f)**	0.877	0.942	0.430	0.907	0.350	0.613	0.73	0.55	0.71
Lactobacillales **(o)**	0.668	0.863	0.756	0.929	0.486	0.682	0.46	0.66	0.57
Leptotrichia	0.283	0.669	0.744	0.931	0.342	0.626	0.69	1.36	1.3
Megasphaera	0.842	0.926	0.580	0.910	0.639	0.781	1.05	0.85	0.99
Neisseria	0.003	0.040	0.214	0.899	0.031	0.251	2.16	2.33	5.38
Oribacterium	0.073	0.261	0.276	0.891	0.233	0.536	0.19	0.52	0.52
Peptostreptococcus	0.386	0.698	0.610	0.917	0.573	0.728	0.26	0.49	0.45
Porphyromonas	0.713	0.883	0.629	0.923	0.313	0.601	1.96	3.29	2.91
Prevotella	0.508	0.816	0.617	0.918	0.071	0.379	17.29	22.05	21.9
Rothia	0.004	0.038	0.208	0.895	0.063	0.345	1.38	0.76	0.48
Streptococcus	0.013	0.085	0.177	0.895	0.146	0.497	51.38	40.15	39.52
TM7 **(p)**	0.432	0.738	0.679	0.922	0.392	0.641	0.16	0.17	0.33
Veillonella	0.096	0.370	0.921	0.959	0.008	0.135	9.55	10.2	8.95

b ALDEx2 analysis was performed on raw read count data; The means shown here are relative abundance (%).

Table S6. Bacterial phyla present in the duodenal mucosal microbiota of all patients. A multivariate analysis was performed including smoking status (Current smoker, previous smoker, or having never smoked), patient diagnosis (functional dyspepsia, iron deficiency, Crohn's disease), age, sex, body mass index (BMI) and proton pump inhibitor (PPI) use. Data were normalised via total sum scaling and centred-log ratio transformation. Significance was determined utilising ANOVA with false discovery rate (FDR) correction.

	**Smoking status**		**Sex**		**PPI use**		**Age**		**BMI**		**Diagnosis**	
**Taxa**	***p*-value**	**FDR**	***p*-value**	**FDR**	***p*-value**	**FDR**	***p*-value**	**FDR**	***p*-value**	**FDR**	***p*-value**	**FDR**
Firmicutes	0.0017	0.01	0.26	0.96	0.48	0.96	0.18	0.91	0.087	0.13	0.28	0.58
Actinobacteria	0.0048	0.015	0.77	0.96	0.96	0.96	0.3	0.91	0.055	0.11	0.76	0.76
Bacteroidetes	0.011	0.021	0.92	0.96	0.89	0.96	0.95	0.95	0.17	0.21	0.11	0.58
Proteobacteria	0.02	0.029	0.83	0.96	0.23	0.96	0.73	0.95	0.023	0.068	0.29	0.58
Fusobacteria	0.072	0.086	0.59	0.96	0.82	0.96	0.95	0.95	0.52	0.52	0.51	0.61
TM7	0.089	0.089	0.96	0.96	0.66	0.96	0.82	0.95	0.0098	0.059	0.4	0.59

Table S7. Bacterial genera present in the duodenal mucosal microbiota of all patients. A multivariate analysis was performed including smoking status (Current smoker, previous smoker, or having never smoked), patient diagnosis (functional dyspepsia, iron deficiency, Crohn's disease), age, sex, body mass index (BMI) and proton pump inhibitor (PPI) use. Data were normalised via total sum scaling and centred-log ratio transformation. Significance was determined utilising ANOVA with false discovery rate (FDR) correction. (p) – phylum; (c) – class; (o) – order; (f) – family.

	**Smoking status**		**Sex**		**PPI use**		**Age**		**BMI**		**Diagnosis**	
**Taxa**	***p*-value**	**FDR**	***p*-value**	**FDR**	***p*-value**	**FDR**	***p*-value**	**FDR**	***p*-value**	**FDR**	***p*-value**	**FDR**
Helicobacter	0.0006	0.011	0.98	0.98	0.16	0.68	0.64	0.73	0.064	0.47	0.39	0.85
Streptococcus	0.0009	0.011	0.078	0.65	0.98	1	0.21	0.59	0.56	0.8	0.74	0.94
Prevotella	0.0015	0.012	0.3	0.9	0.63	1	0.23	0.59	0.51	0.8	0.46	0.85
Veillonella	0.0044	0.027	0.34	0.9	0.79	1	0.35	0.59	0.85	0.92	0.41	0.85
Oribacterium	0.046	0.23	0.64	0.95	0.73	1	0.045	0.38	0.49	0.8	0.32	0.85
Rothia	0.1	0.37	0.76	0.95	0.012	0.23	0.048	0.38	0.71	0.8	0.23	0.84
***Lactobacillales* (o)**	0.1	0.37	0.07	0.65	0.19	0.68	0.43	0.59	0.61	0.8	0.81	0.94
Neisseria	0.19	0.59	0.74	0.95	0.093	0.66	0.23	0.59	0.59	0.8	0.71	0.94
Haemophilus	0.21	0.59	0.94	0.98	0.35	0.88	0.54	0.64	0.49	0.8	0.065	0.79
Fusobacterium	0.28	0.6	0.55	0.92	1	1	0.47	0.59	0.94	0.94	0.98	0.98
***TM7* (p)**	0.3	0.6	0.75	0.95	0.87	1	0.72	0.78	0.0083	0.21	0.38	0.85
Aggregatibacter	0.32	0.6	0.48	0.9	0.15	0.68	0.0065	0.16	0.079	0.47	0.79	0.94
Porphyromonas	0.34	0.6	0.44	0.9	0.58	1	0.84	0.88	0.45	0.8	0.51	0.85
Bulleidia	0.34	0.6	0.14	0.87	0.94	1	0.25	0.59	0.42	0.8	0.23	0.84
Atopobium	0.37	0.6	0.35	0.9	0.11	0.66	0.42	0.59	0.38	0.8	0.95	0.98
Actinomyces	0.38	0.6	0.47	0.9	0.018	0.23	0.39	0.59	0.094	0.47	0.82	0.94
Granulicatella	0.47	0.68	0.98	0.98	0.76	1	0.3	0.59	0.22	0.8	0.27	0.84
***Lachnospiraceae* (f)**	0.5	0.68	0.97	0.98	0.85	1	0.88	0.88	0.27	0.8	0.49	0.85
Leptotrichia	0.53	0.68	0.87	0.98	0.96	1	0.38	0.59	0.091	0.47	0.077	0.79
Peptostreptococcus	0.55	0.68	0.5	0.9	0.94	1	0.13	0.59	0.54	0.8	0.93	0.98
Actinobacillus	0.58	0.68	0.22	0.9	0.76	1	0.061	0.38	0.48	0.8	0.18	0.84
***Gemellales* (o)**	0.62	0.68	0.44	0.9	0.5	1	0.36	0.59	0.7	0.8	0.27	0.84
***Bacilli* (c)**	0.62	0.68	0.44	0.9	0.32	0.88	0.45	0.59	0.69	0.8	0.095	0.79
Megasphaera	0.9	0.9	0.045	0.65	0.9	1	0.28	0.59	0.31	0.8	0.66	0.94
Gemella	0.9	0.9	0.7	0.95	0.23	0.7	0.28	0.59	0.92	0.94	0.82	0.94

Table S8. Bacterial OTUs that were observed to have significantly different relative abundances in the duodenal mucosal microbiota of all patients. Patients were grouped based on smoking status (Current smoker, previous smoker, or having never smoked). Data were normalised via total sum scaling and centred-log ratio transformation, and expressed as mean relative abundance. Significance was determined utilising Kruskal-Wallis with false discovery rate (FDR) correction for multiple comparisons. The numbers in the OTU column represent the Greengenes database reference OTU. Note – When non-normalised (raw) data were analysed for differential relative abundance utilising ALDEx2 (Wilcoxon Rank Test with Benjamini-Hochberg (BH) correction), no significant differences in relative abundance were observed. p-phylum; g-genus; s-species.

OTU	*p*-value (Current vs Never)	FDR *q*-value (Current vs Never)	*p*-value (Current vs Previous)	FDR *q*-value (Current vs Previous)	*p*-value (Previous vs Never)	FDR *q*-value (Previous vs Never)	Current mean[a]	Previous mean[a]	Never mean[a]
p__Firmicutes__g__Streptococcus_1092300*	0.0011	0.028	NS	NS	0.0043	0.038	6.59	5.41	3.32
p__Firmicutes__g__Streptococcus_1088134*	0.0014	0.028	NS	NS	0.0077	0.054	9.04	7.64	5.58
p__Firmicutes__g__Streptococcus_1098340*	0.0017	0.028	NS	NS	0.0049	0.039	11.04	9.59	7.57
p__Actinobacteria__g__Rothia__s__mucilaginosa_368097	0.0017	0.028	NS	NS	NS	NS	5.02	1.24	0.67
p__Firmicutes__g__Streptococcus_1097208	0.0018	0.028	NS	NS	0.011	NS	6.36	4.76	2.95
p__Firmicutes__g__Veillonella__s__dispar_937248	0.0029	0.038	NS	NS	0.0033	0.038	9.05	7.65	6.03
p__Bacteroidetes__g__Prevotella__s__melaninogenica_4307391	NS	NS	NS	NS	0.00037	0.028	2.24	4.89	1.34
p__Bacteroidetes__g__Prevotella__s__melaninogenica_1066621	0.024	NS	NS	NS	0.00072	0.028	6.76	7.43	5.04
p__Firmicutes__g__Veillonella__s__dispar_878104	0.016	NS	NS	NS	0.0015	0.036	5.6	6.03	4.34
p__Firmicutes__g__Veillonella__s__dispar_1019878	0.033	NS	NS	NS	0.0018	0.036	4.97	5.02	2.75
p__Firmicutes__g__Streptococcus_4366889	0.0064	NS	NS	NS	0.0036	0.038	5.15	4.39	2.25
p__Firmicutes__g__Veillonella__s__dispar_New.ReferenceOTU21206	NS	NS	NS	NS	0.0038	0.038	-0.52	3.17	-0.05
p__Firmicutes__g__Veillonella__s__dispar_873174	0.0089	NS	NS	NS	0.0043	0.038	5.72	4.79	3.11

a These means represent relative abundance (%) data that has been centred-log ratio transformed. Statistical analysis was performed on this transformed data. * These OTUs were also significantly different in a multivariate analysis including patient age, sex, BMI, PPI use and diagnosis.

Table S9. *P*-values comparing relative abundances of bacterial genera present in the duodenal mucosal microbiota of either functional dyspepsia (FD) and iron deficiency (ID) patients (Table A), or Crohn's Disease (CD) patients (Table B). Patients were grouped based on smoking status (Current smoker, previous smoker, or having never smoked). Data were normalised via total sum scaling and centred-log ratio transformation, and significance was determined utilising Kruskal-Wallis with false discovery rate (FDR) correction for multiple comparisons. (p) – phylum; (c) – class; (o) – order; (f) – family.

A

FD-ID only						
**Genus**	***p*-value (Current vs Never)**	**FDR *q*-value (Current vs Never)**	***p*-value (Current vs Previous)**	**FDR *q*-value (Current vs Previous)**	***p*-value (Previous vs Never)**	**FDR *q*-value (Previous vs Never)**
Actinobacillus	1	1	1	1	1	1
Actinomyces	0.28	0.78	0.64	1	0.29	1
Aggregatibacter	1	1	1	1	1	1
Atopobium	1	1	1	1	1	1
***Bacilli* (c)**	1	1	1	1	1	1
Bulleidia	1	1	1	1	1	1
Fusobacterium	0.036	0.15	0.044	0.24	1	1
Gemella	0.16	0.5	0.27	0.96	0.69	1
***Gemellales* (o)**	1	1	1	1	1	1
Granulicatella	0.08	0.29	0.41	1	0.18	0.75
Haemophilus	0.77	1	0.65	1	0.56	1
Helicobacter	1	1	1	1	1	1
***Lachnospiraceae* (f)**	1	1	1	1	1	1
***Lactobacillales* (o)**	0.012	0.06	0.11	0.46	0.37	1

Leptotrichia	0.9	1	1	1	0.81	1
Megasphaera	1	1	1	1	1	1
Neisseria	0.45	1	0.47	1	0.88	1
Oribacterium	1	1	1	1	1	1
Peptostreptococcus	1	1	1	1	1	1
Porphyromonas	0.36	0.9	0.94	1	0.051	0.6
Prevotella	0.00022	0.0028	0.043	0.24	0.072	0.6
Rothia	0.0031	0.019	0.049	0.24	0.13	0.65
Streptococcus	0.0015	0.012	0.018	0.22	0.12	0.65
***TM73* (p)**	1	1	1	1	1	1
Veillonella	0.0002	0.0028	0.01	0.22	0.065	0.6

B

**CD only**						
**Genus**	***p*-value (Current vs Never)**	**FDR *q*-value (Current vs Never)**	***p*-value (Current vs Previous)**	**FDR *q*-value (Current vs Previous)**	***p*-value (Previous vs Never)**	**FDR *q*-value (Previous vs Never)**
Actinobacillus	1	1	1	1	0.53	1
Actinomyces	0.53	1	0.18	0.75	0.051	0.21
Aggregatibacter	1	1	1	1	1	1
Atopobium	1	1	1	1	0.73	1
***Bacilli* (c)**	1	1	1	1	1	1
Bulleidia	1	1	1	1	1	1

Fusobacterium	0.63	1	0.8	1	0.53	1
Gemella	0.37	1	1	1	0.37	1
***Gemellales* (o)**	0.53	1	1	1	1	1
Granulicatella	0.73	1	0.32	1	1	1
Haemophilus	0.1	1	0.13	0.75	0.0012	0.03
Helicobacter	1	1	1	1	1	1
***Lachnospiraceae* (f)**	1	1	1	1	1	1
***Lactobacillales* (o)**	0.53	1	0.21	0.75	0.022	0.11
Leptotrichia	0.45	1	1	1	1	1
Megasphaera	0.45	1	1	1	0.84	1
Neisseria	1	1	0.75	1	0.84	1
Oribacterium	1	1	1	1	1	1
Peptostreptococcus	1	1	1	1	1	1
Porphyromonas	0.45	1	0.21	0.75	0.1	0.36
Prevotella	0.45	1	0.097	0.75	0.022	0.11
Rothia	0.29	1	1	1	1	1
Streptococcus	0.37	1	0.13	0.75	0.014	0.11
***TM73* (p)**	1	1	1	1	1	1
Veillonella	0.63	1	0.073	0.75	0.014	0.11
