# Supplementary material for: Influence of cigarette smoking on the human duodenal mucosa-associated microbiota
Source: Microbiome. 2018 Aug 29;6:150. doi: 10.1186/s40168-018-0531-3 (PMC6116507; doi:10.1186/s40168-018-0531-3)
Supplement: Supplementary file 2 — Supplementary Methods. (PDF 163 kb) [file 40168_2018_531_MOESM2_ESM.pdf]

SUPPLEMENTARY METHODS

INFLUENCE OF CIGARETTE SMOKING ON THE HUMAN DUODENAL MUCOSA ASSOCIATED MICROBIOTA

Erin R. Shanahan[1,2,3#], Ayesha Shah[1,2], Natasha Koloski[1,4], Marjorie Walker[4], Nicholas J Talley[4], Mark Morrison[2,3*], and Gerald J Holtmann[1,2*]

1. Department of Gastroenterology and Hepatology, Princess Alexandra Hospital, and Faculty of Medicine, The University of Queensland, Woolloongabba, Queensland, Australia
2. Translational Research Institute, Woolloongabba, Queensland, Australia
3. The University of Queensland Diamantina Institute, Faculty of Medicine, The University of Queensland, St Lucia, Queensland, Australia
4. Faculty of Health and Medicine, University of Newcastle, Newcastle, New South Wales, Australia

[#] Current address: School of Life and Environmental Sciences, Charles Perkins Centre, The University of Sydney, Camperdown, New South Wales, Australia

Bioinformatics Workflow

Sequence data was processed using the Quantitative Insights into Microbial Ecology (QIIME) pipeline (version 1.9.1) [1].

The following steps were performed in the QIIME Virtual Box on demultiplexed fastq files:

Split Libraries using a Phred quality threshold of Q20

```
split_libraries_fastq.py -i [list fastq file names] --sample_id [list
sample IDs] --barcode_type not-barcoded -q 20 -o split_for_reads/ -m
map.txt
```

The following steps were performed using QIIME as implemented in Bio-Linux (version 8; http://environmentalomics.org/bio-linux/) via the University of Queensland High Performance Computing Cluster:

Assign Operational Taxonomic Units (OTUs) via open reference picking [2], using the “seq.fna” file generated from the split libraries step. The Greengenes database (version 13.8) was used as the reference database and a sequence similarity of 97% applied [3].

```
pick_open_reference_otus.py -i $TMPDIR/seqs.fna -o $TMPDIR/output/ -r
/usr/share/qiime/data/gg_13_8_otus/rep_set/97_otus.fasta -s 0.1 -a -O 6
```

The resulting OTU table was chimera-checked using ChimeraSlayer [4].

```
parallel_identify_chimeric_seqs.py -i
$TMPDIR/output/pynast_aligned_seqs/rep_set_aligned.fasta -m ChimeraSlayer -
o $TMPDIR/output/chimeric_seqs.txt -a
/usr/share/qiime/data/gg_13_8_otus/rep_set/97_otus.fasta -O 6
```

The following steps were performed in the QIIME Virtual Box following OTU picking:

Filter OTU table to remove sequences not classified as Bacteria or Archaea. The output of the OTU picking step was used (no chimeric sequences were identified thus this table was used directly).

```
filter_taxa_from_otu_table.py -i
otu_table_mc2_w_tax_no_pynast_failures.biom -o otu_table_micro.biom -p
k__Bacteria,k__Archaea
```

Filter the resultant table to remove sequences with a relative abundance of less than 0.1%.

```
filter_otus_from_otu_table.py -i otu_table_micro.biom -o
otu_table_micro_filtered.biom --min_count_fraction 0.001
```

Filter to generate table with control samples only.

```
filter_samples_from_otu_table.py -i otu_table_micro_filtered.biom -o
otu_table_micro_filtered_controls.biom --sample_id_fp
list_control_samples.txt
```

```
biom convert -i otu_table_micro_filtered_controls.biom -o
otu_table_micro_filtered_controls.txt --to-tsv --header-key taxonomy
```

Generate a list of specific “contaminant” OTUs present in the control samples (See “List of contaminant OTUs” below). Filter these specific OTUs from the small intestinal samples to generate an OTU table representing contamination free small intestinal sequences.

```
filter_otus_from_otu_table.py -i otu_table_micro_filtered.biom -o
otu_table_micro_filtered_nocontam.biom –e contam_otu_list.txt
```

```
filter_samples_from_otu_table.py -i otu_table_micro_filtered_nocontam.biom
-o otu_table_micro_filtered_controls_nocontam_samples.biom --sample_id_fp
list_control_samples.txt --negate_sample_id_fp
```

Exclude all samples with a final read count of less than 1000 sequence reads from the OTU table.

```
filter_samples_from_otu_table.py -i
otu_table_micro_filtered_nocontam_samples.biom -o otu_table_final.biom –n
1000
```

```
biom convert -i otu_table_final.biom -o otu_table_final.txt --to-tsv --
header-key taxonomy
```

Diversity Analysis

The following steps were performed in the QIIME Virtual Box on the final OTU table:

Perform multiple rarefactions (randomly subsample OTU table 100 times at a depth of 1000 reads)

```
multiple_rarefactions_even_depth.py -i otu_table_final.biom -o
rarefied_otu_tables/ -d 1000 -n 100
```

Average out the rarefied results – sum together all the OTU tables and average the results (export biom table and divide all counts by 100 after merging tables).

```
merge_otu_tables.py -i [list all 100 rarefied out tables] –o
out_table_rare1000_merge.biom
```

```
biom convert -i otu_table_rare1000_merge.biom -o
otu_table_rare1000_merge.txt --to-tsv --header-key taxonomy
```

After generating the averaged rarefied OTU table, convert back to biom format.

```
biom convert -i otu_table_rare1000_merge_average.txt -o
out_table_rare1000_final.biom --to-hdf5 --table-type="OTU table" --process-
obs-metadata taxonomy
```

Perform alpha-diversity analysis.

```
alpha_rarefaction.py -i otu_table_rare1000_final.biom -o alpha_div/ -t
rep_set.tre -m map.txt -e 1000 –a –O 2
```

Perform beta-diversity analysis.

```
beta_diversity_through_plots.py -i otu_table_rare1000_final.biom -o
beta_div/ -t rep_set.tre -m map.txt –e 1000 –a –O 2
```

Statistical Analysis

Statistical analysis on alpha diversity data

Alpha diversity values for the Chao1 and phylogenetic diversity metrics (generated as above via the alpha rarefaction command) were exported from QIIME and imported into the statistical software Prism. The Kruskal-Wallis test was applied to determine significance between patient groups.

Statistical analysis on beta diversity data

The distance matrix (generated as above via the beta diversity command) for the weighted Unifrac metric was exported from QIIME and uploaded to the online microbiome analysis tool Calypso (http://cgenome.net/wiki/index.php/Calypso) [5], along with a mapping file (containing patient/sample metadata) and the corresponding OTU table (otu_table_final.biom, generated as above). The ADONIS metric was implemented, adjusting for sex, age, body mass index (BMI), proton pump inhibitor (PPI) use and diagnosis.

Analysis of relative abundance

A mapping file (containing patient/sample metadata) and the corresponding OTU table (otu_table_final.biom, generated as above) were uploaded to the online microbiome analysis tool Calypso (http://cgenome.net/wiki/index.php/Calypso) [5]. Data was normalised using this program via total sum scaling and then centred-log ratio transformation. Significant differences between patient groups were assessed using Kruskal-Wallis (KW) with False Discovery Rate (FDR) correction for multiple comparisons. Linear discriminant analysis effect size (LEfSe) was also performed on this data. In addition, the raw read count data (directly from uploaded biom file) was analysed using the ALDEx2 function (Wilcoxon test with Benjamini-Hochberg corrected p value).

Multivariate model generation

The final OTU table was filtered to include only samples representing current or never smokers, and only patients with FD and/or ID. This table, along with a mapping file (containing patient/sample metadata) were uploaded to the Mixomics [6] web interface available through the University of Queensland/Queensland Facility for Advanced Bioinformatics (QFAB) (mixomics.qfab.org). The OTU table was normalised via total sum scaling and then centred-

log ratio transformation. Sparse partial least squares discriminant analysis (sPLS-DA) was performed using default settings (via the "tune" and "splsda" functions), with the model validated using leave-one-out cross validation (repeated 30 times). This model was then tested on a second data set. Specifically, the final OTU table was filtered to include only samples representing current or never smokers, and only patients with CD. The OTU table was normalised via total sum scaling and then centred-log ratio transformation. The "predict" function was used to classify the patients in regards to smoking status within the CD cohort, based on the model generated with the FD-ID cohort.

Batch Checking

The Illumina sequencing for this study was performed across 2 separate sequencing runs (with identical methods used for both; at the same facility). The generated data was processed as a single complete dataset during all bioinformatics procedures. A principal coordinate plot was generated to test for any batch effect resulting from the sequencing runs. As per the figure below, there was no significant difference between the two sequencing runs (ADONIS p=0.2). Open circles: sequencing run 1; orange circles, sequencing run 2.

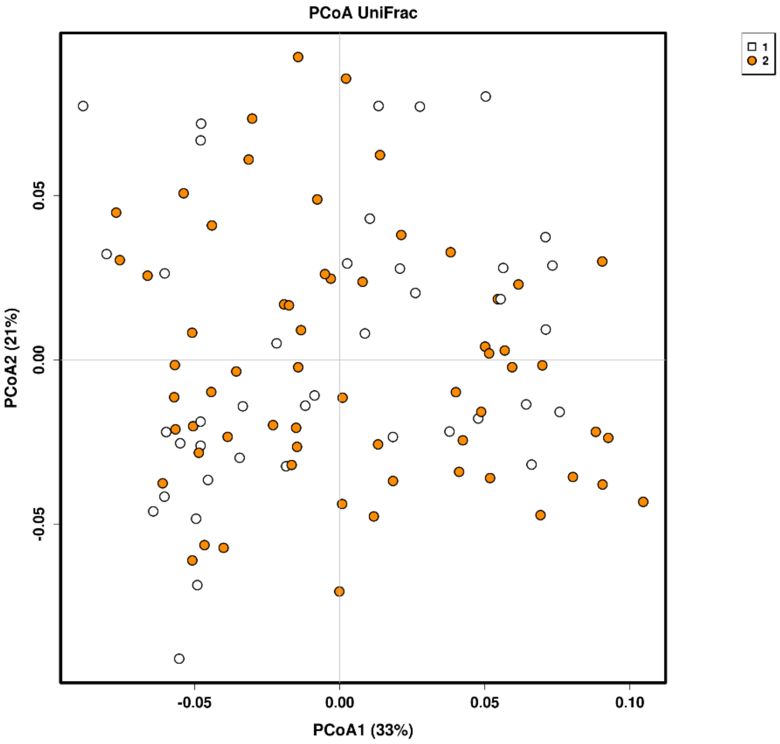

List of contaminant OTUs

This list was generated from the out_table_micro_filtered_controls.txt table, in which any OTU observed in at least one control sample, at a relative abundance of greater than 1% on average across the reagent controls, or greater than 2% relative abundance in at least two reagent controls was included. This criteria ensured all significant contaminants could be removed from the patient samples. A total of 18 reagent control samples were included for the study.

List of contaminant OTUs removed from OTU table:

OTU (Greengenes Number)	Taxonomy
179312	k__Bacteria; p__Actinobacteria; c__Actinobacteria; o__Actinomycetales; f__Cellulomonadaceae; g__Cellulomonas
4481506	k__Bacteria; p__Bacteroidetes; c__Bacteroidia; o__Bacteroidales; f__S24-7
334761	k__Bacteria; p__Bacteroidetes; c__Bacteroidia; o__Bacteroidales; f__S24-7
346267	k__Bacteria; p__Bacteroidetes; c__Bacteroidia; o__Bacteroidales; f__S24-7
442846	k__Bacteria; p__Bacteroidetes; c__Bacteroidia; o__Bacteroidales; f__S24-7
224486	k__Bacteria; p__Cyanobacteria; c__Oscillatoriophycideae
New.ReferenceOTU26271	k__Bacteria; p__Firmicutes; c__Bacilli; o__Bacillales; f__Paenibacillaceae; g__Paenibacillus
1090059	k__Bacteria; p__Firmicutes; c__Bacilli; o__Bacillales; f__Staphylococcaceae; g__Staphylococcus
1111582	k__Bacteria; p__Firmicutes; c__Bacilli; o__Lactobacillales; f__Streptococcaceae
354097	k__Bacteria; p__Firmicutes; c__Clostridia; o__Clostridiales
1105860	k__Bacteria; p__Firmicutes; c__Erysipelotrichi; o__Erysipelotrichales; f__Erysipelotrichaceae; g__Allobaculum
274912	k__Bacteria; p__Firmicutes; c__Erysipelotrichi; o__Erysipelotrichales; f__Erysipelotrichaceae; g__Allobaculum
New.ReferenceOTU38018	k__Bacteria; p__Firmicutes; c__Erysipelotrichi; o__Erysipelotrichales; f__Erysipelotrichaceae; g__Allobaculum
558740	k__Bacteria; p__Proteobacteria; c__Alphaproteobacteria; o__Rhizobiales; f__Bradyrhizobiaceae; g__Afipia
788519	k__Bacteria; p__Proteobacteria; c__Betaproteobacteria; o__Burkholderiales; f__Alcaligenaceae

1061429	k__Bacteria; p__Proteobacteria; c__Betaproteobacteria; o__Burkholderiales; f__Comamonadaceae
816420	k__Bacteria; p__Proteobacteria; c__Betaproteobacteria; o__Burkholderiales; f__Comamonadaceae
1024520	k__Bacteria; p__Proteobacteria; c__Betaproteobacteria; o__Burkholderiales; f__Comamonadaceae; g__Comamonas
335466	k__Bacteria; p__Proteobacteria; c__Betaproteobacteria; o__Burkholderiales; f__Oxalobacteraceae
341936	k__Bacteria; p__Proteobacteria; c__Betaproteobacteria; o__Burkholderiales; f__Oxalobacteraceae
783719	k__Bacteria; p__Proteobacteria; c__Betaproteobacteria; o__Burkholderiales; f__Oxalobacteraceae; g__Ralstonia
510057	k__Bacteria; p__Proteobacteria; c__Gammaproteobacteria; o__Pseudomonadales
1097359	k__Bacteria; p__Proteobacteria; c__Gammaproteobacteria; o__Pseudomonadales; f__Moraxellaceae; g__Acinetobacter; s__lwoffii
1041394	k__Bacteria; p__Proteobacteria; c__Gammaproteobacteria; o__Pseudomonadales; f__Pseudomonadaceae; g__Pseudomonas
928406	k__Bacteria; p__Proteobacteria; c__Gammaproteobacteria; o__Pseudomonadales; f__Pseudomonadaceae; g__Pseudomonas
560075	k__Bacteria; p__Proteobacteria; c__Gammaproteobacteria; o__Pseudomonadales; f__Pseudomonadaceae; g__Pseudomonas
912967	k__Bacteria; p__Proteobacteria; c__Gammaproteobacteria; o__Pseudomonadales; f__Pseudomonadaceae; g__Pseudomonas
928829	k__Bacteria; p__Proteobacteria; c__Gammaproteobacteria; o__Pseudomonadales; f__Pseudomonadaceae; g__Pseudomonas
818369	k__Bacteria; p__Proteobacteria; c__Gammaproteobacteria; o__Pseudomonadales; f__Pseudomonadaceae; g__Pseudomonas
780261	k__Bacteria; p__Proteobacteria; c__Gammaproteobacteria; o__Pseudomonadales; f__Pseudomonadaceae; g__Pseudomonas
New.ReferenceOTU1025	k__Bacteria; p__Proteobacteria; c__Gammaproteobacteria; o__Pseudomonadales; f__Pseudomonadaceae; g__Pseudomonas
1083508	k__Bacteria; p__Proteobacteria; c__Gammaproteobacteria; o__Xanthomonadales; f__Xanthomonadaceae; g__Stenotrophomonas

References (Supplementary Methods)

1. Caporaso JG, Kuczynski J, Stombaugh J, et al. QIIME allows analysis of high-throughput community sequencing data. Nature methods 2010;**7**(5):335-6.
2. Rideout JR, He Y, Navas-Molina JA, et al. Subsampled open-reference clustering creates consistent, comprehensive OTU definitions and scales to billions of sequences. PeerJ 2014;**2**:e545.
3. DeSantis TZ, Hugenholtz P, Larsen N, et al. Greengenes, a chimera-checked 16S rRNA gene database and workbench compatible with ARB. Applied and environmental microbiology 2006;**72**(7):5069-72.
4. Haas BJ, Gevers D, Earl AM, et al. Chimeric 16S rRNA sequence formation and detection in Sanger and 454-pyrosequenced PCR amplicons. Genome Res 2011;**21**(3):494-504.
5. Zakrzewski M, Proietti C, Ellis JJ, et al. Calypso: a user-friendly web-server for mining and visualizing microbiome-environment interactions. Bioinformatics 2017;**33**(5):782-83.
6. Le Cao KA, Costello ME, Lakis VA, et al. MixMC: A Multivariate Statistical Framework to Gain Insight into Microbial Communities. PloS one 2016;**11**(8):e0160169.
